# Design and Synthesis of Thiourea-Conjugating Organic Arsenic D-Glucose with Anticancer Activities

**DOI:** 10.3390/molecules29122850

**Published:** 2024-06-14

**Authors:** Boqiao Fu, Wenxuan Liu, Yufeng Wang, Guorui Li, Yingsha Wang, Xinyuan Huang, Hongan Shi, Caiqin Qin

**Affiliations:** 1 College of Chemistry and Materials Science, Hubei Engineering University, Xiaogan 432000, China; 18054446487@163.com (W.L.); xiaoao67356320@163.com (Y.W.); qincq@hbeu.edu.cn (C.Q.); 2 Hunan Provincial Key Laboratory of the Research and Development of Novel Pharmaceutical Preparations, the “Double-First Class” Application Characteristic Discipline of Hunan Province (Pharmaceutical Science), Changsha Medical University, Changsha 410219, China; wudalgr@aliyun.com; 3 State Key Laboratory for Chemo/Bio-Sensing and Chemometrics, College of Chemistry and Chemical Engineering, School of Biomedical Sciences, Hunan University, Changsha 410082, China; wangys@hnu.edu.cn; 4 Hubei Key Laboratory of Quality Control of Characteristic Fruits and Vegetables, College of Life and Technology, Hubei Engineering University, Xiaogan 432000, China; huangxy5885@163.com (X.H.); shihongan1991@163.com (H.S.)

**Keywords:** (1,3,2-dithiarsinan-2-yl) aniline, thiourea, 2,3,4,6-*O*-tetra acetyl-α-d-glucopyranose, anticancer, HCT-116

## Abstract

Organic arsenic compounds such as *p*-aminophenylarsine oxide (*p*-APAO) are easier for structural optimization to improve drug-like properties such as pharmacokinetic properties, therapeutic efficacy, and target selectivity. In order to strengthen the selectivity of 4-(1,3,2-dithiarsinan-2-yl) aniline 7 to tumor cell, a thiourea moiety was used to strengthen the anticancer activity. To avoid forming a mixture of α/β anomers, the strategy of 2-acetyl’s neighboring group participation was used to lock the configuration of 2,3,4,6-tetra-*O*-acetyl-β-d-glucopyranosyl isothiocyanate from 2,3,4,6-tetra-*O*-acetyl-α-d-glucopyranosyl bromide. 1-(4-(1,3,2-dithiarsinan-2-yl) aniline)-2-N-(2,3,4,6-tetra-*O*-acetyl-β-d-glucopyranos-1-yl)-thiourea 2 can increase the selectivity of human colon cancer cells HCT-116 (0.82 ± 0.06 μM vs. 1.82 ± 0.07 μM) to human embryonic kidney 293T cells (1.38 ± 0.01 μM vs. 1.22 ± 0.06 μM) from 0.67 to 1.68, suggesting a feasible approach to improve the therapeutic index of arsenic-containing compounds as chemotherapeutic agents.

## 1. Introduction

Arsenic trioxide (As, +III) and its derivatives have been used for various purposes for more than 2000 years [1,2,3]. In the 1970s, arsenic trioxide was used to heal acute promyelocytic leukemia (APL) in clinical application by Zhang Tingdong [1]. Subsequently, arsenic trioxide (Trade Name: Trisenox) has been approved for the treatment of relapsed or refractory APL by the U.S. Food and Drug Administration since 2000 and European Medicines Agency since 2017, respectively. The mechanisms of action of arsenic trioxide have revealed that the drug is able to promote the catabolic degradation of an oncogenic fusion protein [4,5,6], disrupt the mitochondrial function [7], downregulate Bcl-2 expression [8], and reactivate mutant p53 for tumor suppression [9]. Despite the remarkable success of arsenic trioxide in the treatment of APL, limitations of inorganic arsenic compound as a chemotherapeutic was the systemic toxicity, associated with its poor pharmacokinetic properties. This may be attributed to the rapid renal clearance of arsenic trioxide metabolites [2,10].

**Figure 1:**
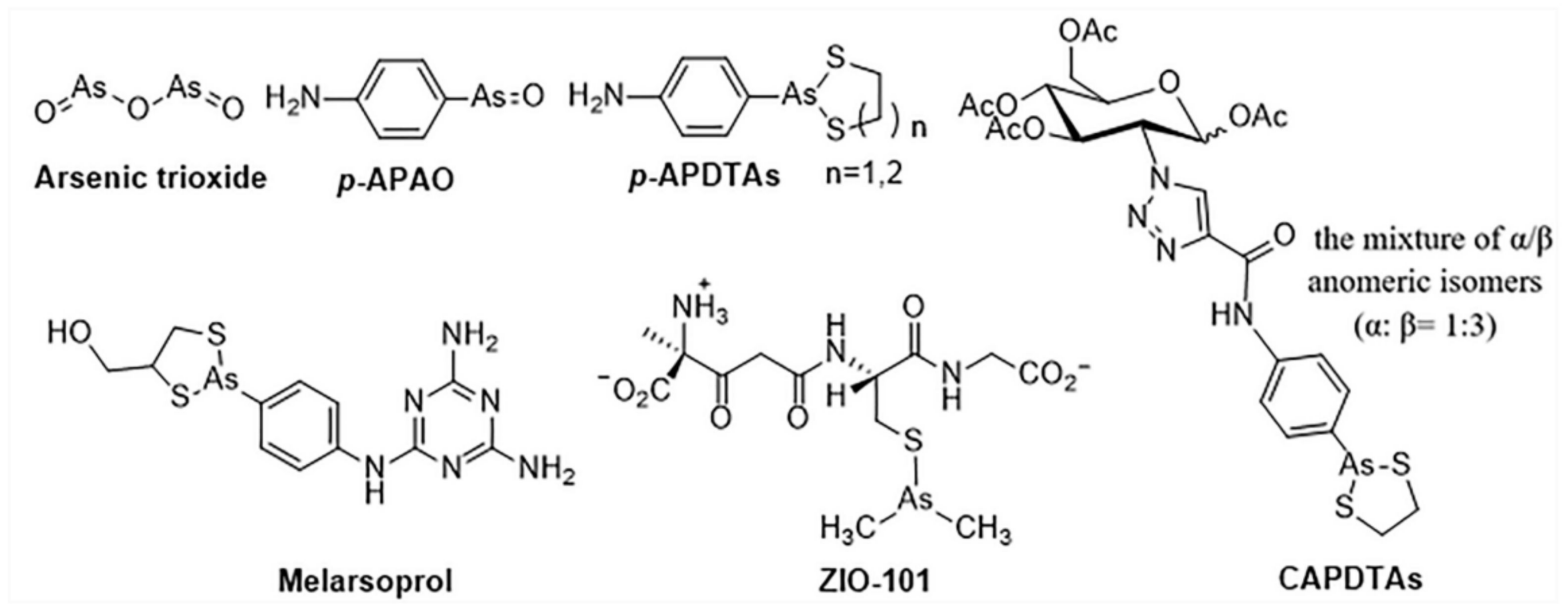
Representative arsenic-containing compounds with anticancer activities [3,18].

In comparison with inorganic arsenic compounds, organic arsenic compounds are easier for structural optimization to improve drug-like properties such as pharmacokinetic properties, therapeutic efficacy, and target selectivity. Organic arsenic compounds—*p*-aminophenylarsine oxide (*p*-APAO) [4,11,12] and others [3,13,14,15]—were investigated either as preclinical and clinical experimental drugs or as molecular probes in cancer cells. Arsenic sulfide and its derivatives showed antitumor activity in solid tumor cell lines such as HCT 116 [16,17,18] (Figure 1).

Urea cycle dysregulation (UCD) in cancer is a prevalent phenomenon in multiple cancers. It is also associated with a worse prognosis but a better response to immune therapy [19]. Small organic molecules with a thiourea moiety have been widely used in the treatment of anticancer [20,21,22,23].

Combined with the previous basis of the design and synthesis of small molecules containing urea structure and based on the principle of bioisosterism, we proposed the research topic of the design, synthesis and bioactivity of small-molecule compounds containing a thiourea structure. Here, the moiety of thiourea-conjugating organic arsenic was induced to anomeric carbon of D-glucose derivatives.

**Figure 2:**
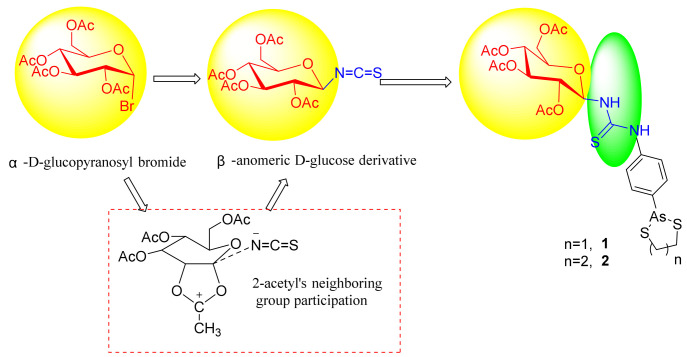
Design of target compounds **1** and **2**.

Based on the derivatives of D-glucosamine, it could reduce the toxicity of trivalent arsenic compound and other pharmacophore [24,25]. Actually these D-glucosamine linker trivalent arsenic compounds and other pharmacophore a the mixture of α/β anomeric isomers in dynamic equilibrium. To avoid the effect of enantiomer on biological activity, here, a novel glucose conjugated arsenic compound was designed as the follows: thiourea was used to strengthen the anticancer biological activities; the anomeric group’s steric configuration of D-glucosamine derivative was locked by the reaction of 2-acetyl’s neighboring group participation via five-membered glycosyl oxocarbenium ion (Figure 2) to avoid the effect of enantiomer on biological activity [25,26]. 2,3,4, 6-tetra-*O*-acetyl-β-d-glucopyranosyl isothiocyanate was designed as precursor of 1-amine-β-d-glucose to avoid the effect of enantiomer on biological activity. Isothiocyanate’s strong electrophilicity was used to react with amine in compound 6 or 7 to form derivatives of 1-amine β-d-glucose 1 and 2, respectively.

## 2. Results and Discussion

**Scheme 1:**
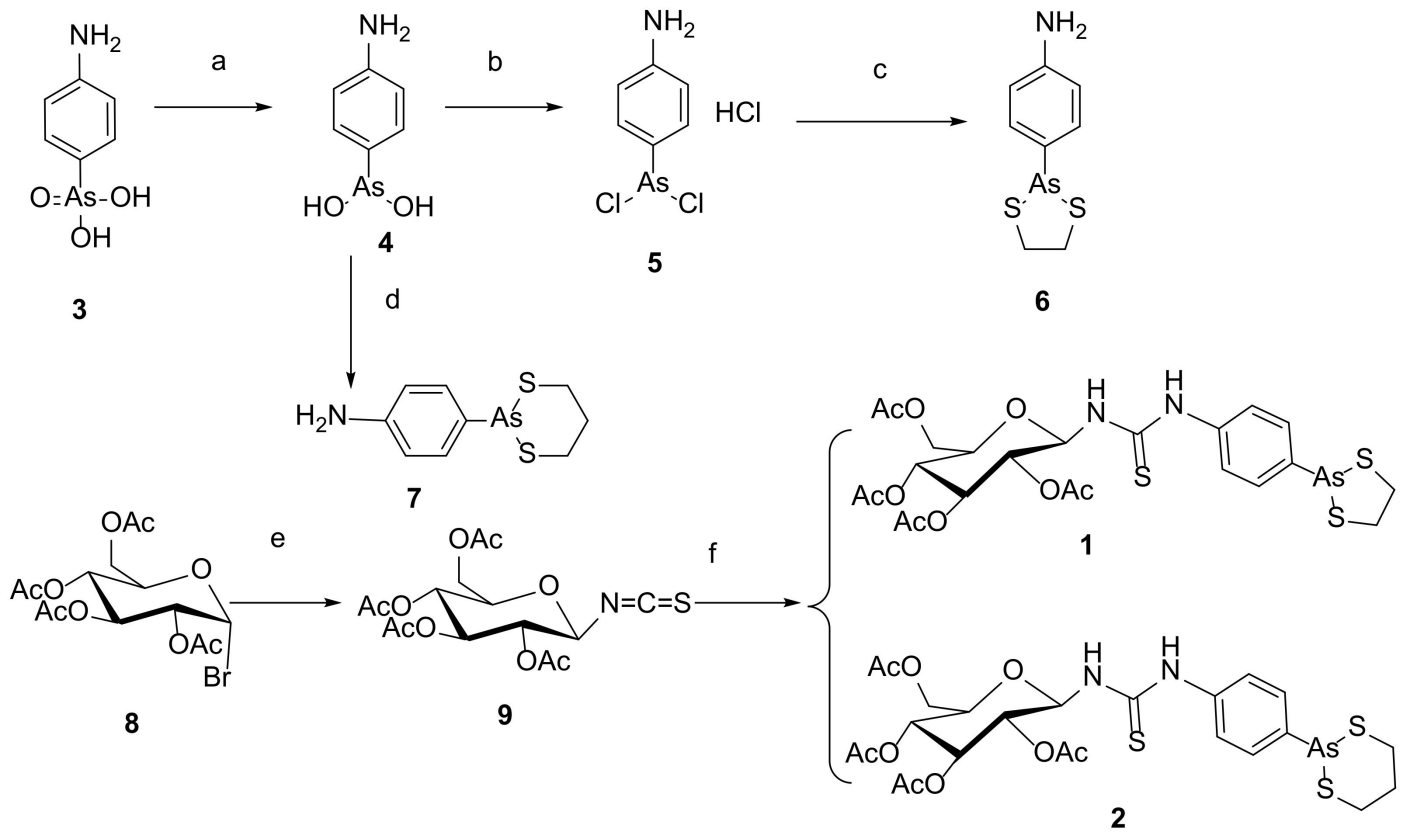
Reaction conditions: (**a**) SO_2_, KI, HCl, MeOH; (**b**) HCl(c); (**c**) ethane-1,2-dithiol, NaHCO_3_, MeOH, 82%; (**d**) ethanol, propane-1,3-dithiol, reflux; (**e**) (i) KSCN, 4 Å MS, (n-Bu)_4_NBr, CH_3_CN, r.t. for 4 h; (ii) 8, N_2_, reflux for 4 h; (**f**) **6**, CH_2_Cl_2_, r.t. overnight, 81.9% for **1**; **7**, CH_2_Cl_2_, r.t. overnight, 71.5% for **2**.

**Figure 3:**
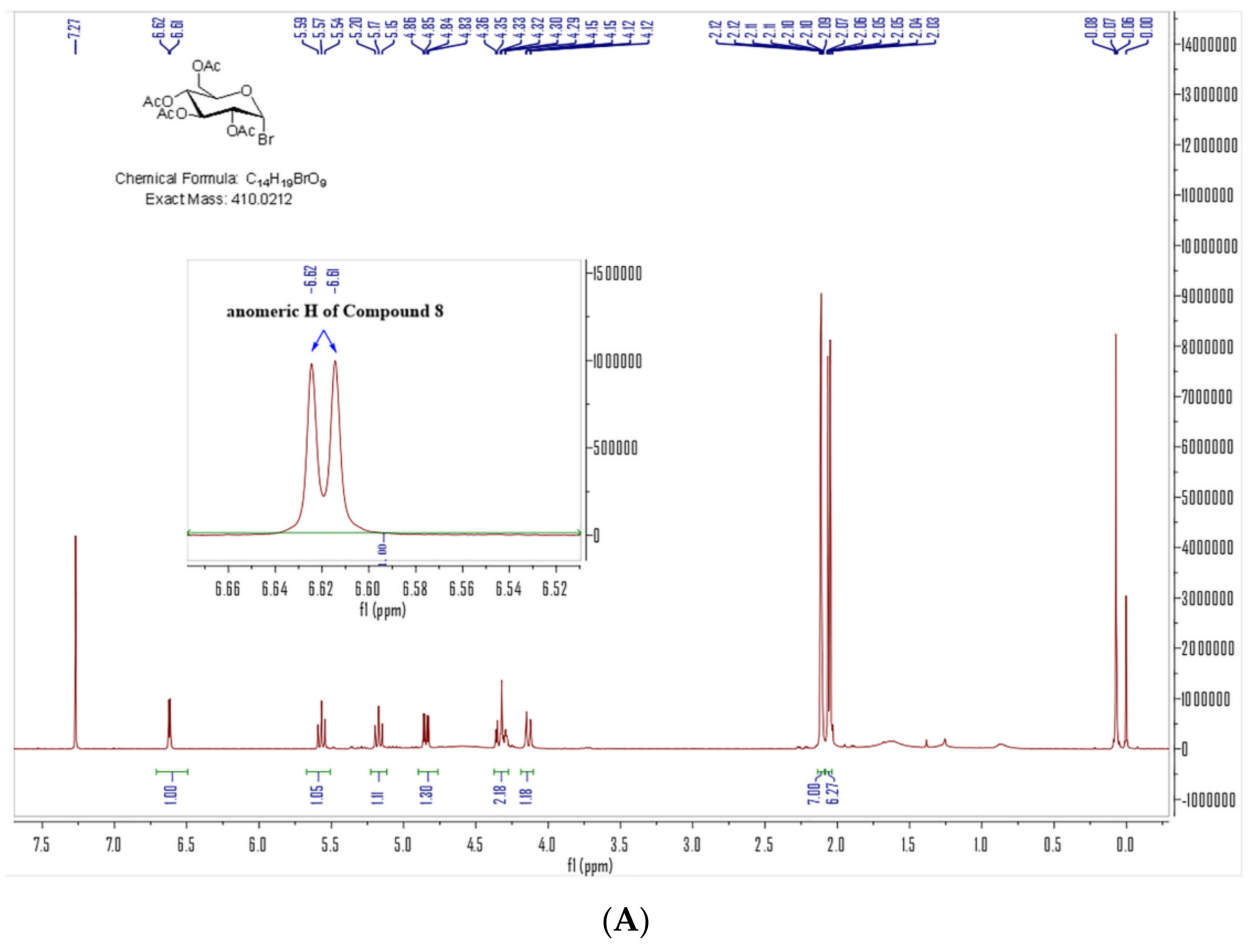

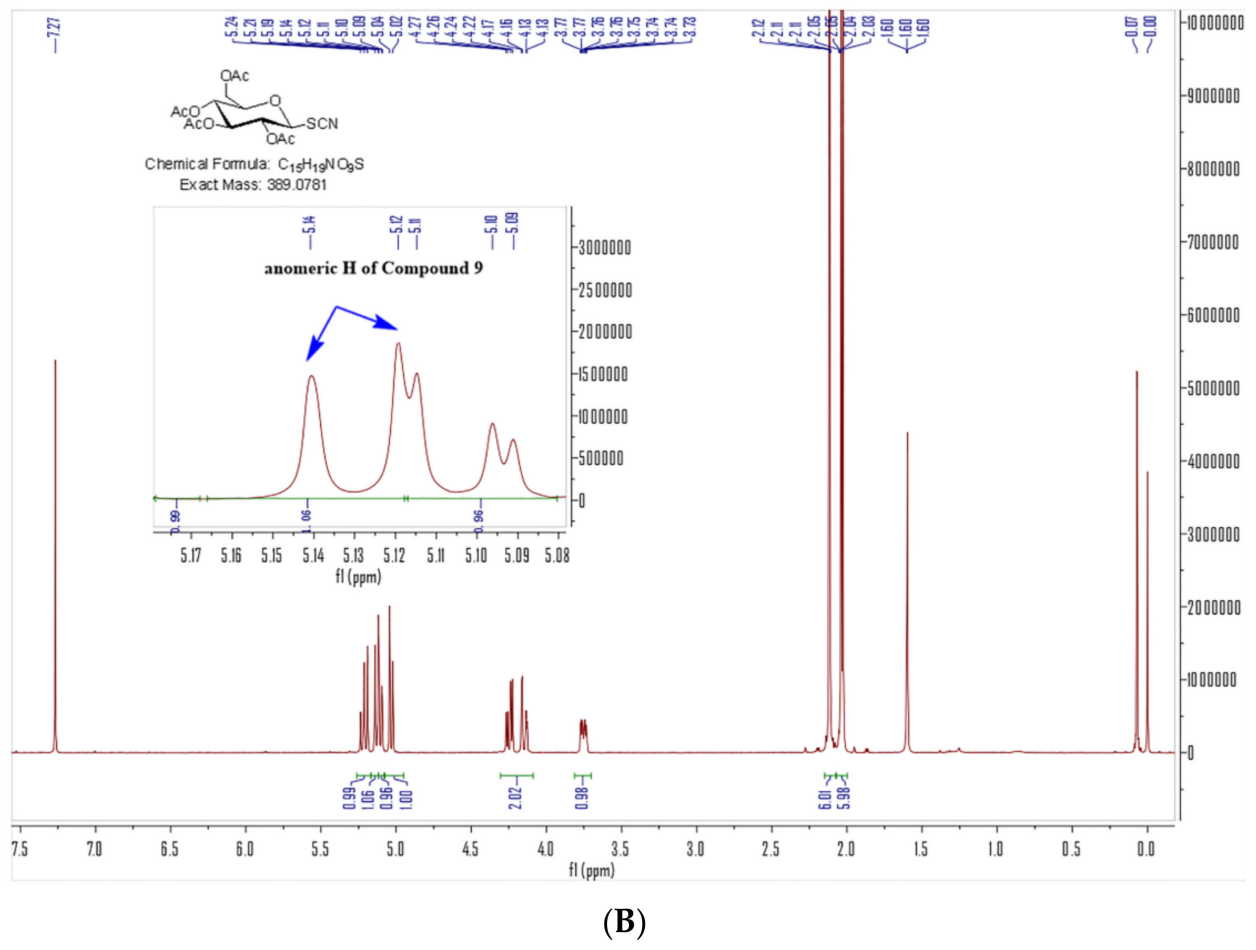
^1^H NMR of Compound **8** (**A**) and **9** (**B**) (400 MHz, CDCl_3_) (Blue arrows are anomeric H).

Our target compounds, the derivatives of 4-aminophenylarsenous acid (As, +III) linking to the anomer of D-glucose to form β-configuration D-glucose-arsenate compounds were designed as follows: the synthesis routes were started from commercial chemical 4-aminophenylarsonic acid (As, +V) **3**. It was reduced to 4-aminophenylarsenic acid (As, +III) **4** by sulfur dioxide under a catalyst of minor iodine. The solution of **4** in ethanol was heated to reflux with propane-1,3-dithiol to obtain compound **7** [27,28,29]. Compound **4** was also dissolved in concentrated HCl to produce intermediate **5**. It further reacted with ethane-1,2-dithiol at room temperature to obtain compound **6** under aqueous sodium carbonate [18]. 2,3,4,6-*O*-tetraacetyl-α-D-glucopyranosyl bromide **8** reacted with potassium thiocyanate through nucleophilic substitution to form β-anomer of D-glucose derivative **9** under the catalyst of a 4 Å molecular sieve and (n-Bu)_4_NBr as a phase transfer catalyst. With the aid of 2-acetyl’s neighboring group participation, the configuration of SCN formed the β-anomer via a five-membered glycosyl oxocarbenium ion to replace the original α-anomer [30]. The chemical shift of anomeric H in compound **8** is 6.62–6.61 ppm. *J*_1,2_ (coupling constant) of anomeric H of compound **8** is 4.0 Hz (Figure 3A), which is in the *J*_1,2_ range of α-D-glucose (3–5 Hz), while the peak at 5.14–5.12 ppm is anomeric H of compound **9**. Anomeric H’s *J*_1,2_ (coupling constant) of compound **9** is 8.0 Hz, which is in the *J*_1,2_ range of β-D-glucose (6–9 Hz) in ^1^H NMR (Figure 3B). The anomeric H’s coupling constants (*J*_1,2_) of compound **1** and **2** are 8.0 Hz and 8.0 Hz, respectively. They are also in the anomeric H’s *J*_1,2_ of β-D-glucose (6–9 Hz) range in ^1^H NMR [31]. These results showed that the anomeric H has been changed from α configuration (compound **8**) to β one (compound **9**, **1** and **2**). In the following steps, compounds **6** and **7** reacted with **9** involving a nucleophilic attack to reach our target compounds **1** and **2** at room temperature with yields of 81.9% and 71.5%, respectively (Scheme 1).

The synthesized compounds were evaluated for their antiproliferation activities against Hela, HepG2, HCT-116 and 293T via a MTT assay [25]. Hela, HepG2 and HCT-116 were chosen as solid tumor cells. 293T was used as a normal cell. The cytotoxicity results shown in Table 1 indicated that compound **2** with a six-membered ring of 1,3,2-dithiarsinane (As, +III) modified has higher cytotoxicity activities on Hela (11.05 μM vs. 14.4 μM), HepG2 cells (2.81 μM vs. 7.62 μM) and HCT-116 (0.82 μM vs. 1.77 μM) than five-membered ring of 1,3,2-dithiarsolane (As, +III) modified compound **1**. Similarly, compound **7** has better anticancer activities than compound **6**. 

**Figure 4:**
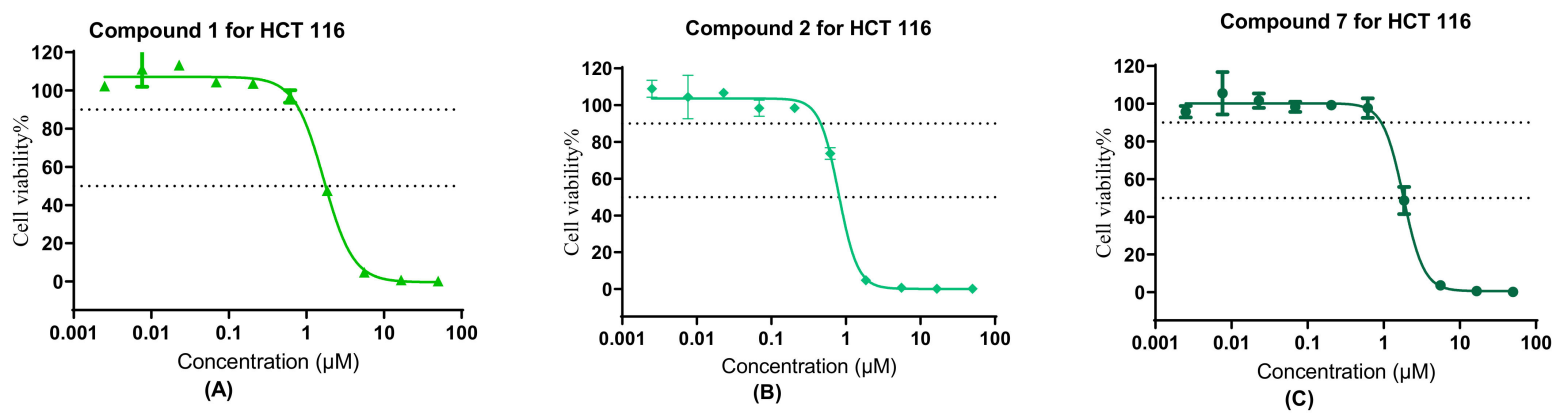
Cytotoxicity of compound **1** (**A**), **2** (**B**) and **7** (**C**) in HCT-116 cells measured by the MTT assay (means ± SD).

Compounds **1**, **2**, and **6** showed some selectivity against HCT-116 cells over 293T cells (in Table 1); among them, 293T cells were chosen as normal cells, except for compound **7**. The cytotoxicity of compounds **1**, **2**, **6** and **7** on 293T was generally maintained at the same level. In comparison with non-2,3,4,6-*O*-tetraacetyl-β-D-glucopyranosyl modification compound **7**, the 2,3,4,6-*O*-tetraacetyl-β-D-glucopyranosyl modification compound **2** showed that the selectivity index (HCT-116 over 293T) was increased by a factor of 1.5. On the contrary, the selectivity index of compound **1** reduced, compared with that of non-2,3,4,6-*O*-tetraacetyl-β-D-glucopyranosyl modification compound **6** (1.77 vs. 2.18) (Figure 4).

## 3. Materials and Methods

All the chemical reagents and solvents were purchased from Sinopharm Group Company limited (Shanghai, China). They were used without further purification, unless specified otherwise. 4-Aminophenylarsonic acid was bought from Tokyo Chemical Industry (Shanghai) Development Co., Ltd. (Shanghai, China). All anhydrous reactions were performed under nitrogen atmosphere. Organic phases were dried over anhydrous Na_2_SO_4_ and removed under reduced pressure during work-up.

Purities of the intermediates were established by silica gel (200–300 mesh) column chromatography. Thin-layer chromatography (TLC) was carried out by silica gel GF254, both of which were obtained from Qingdao Ocean Chemicals (Qingdao, China). In all experiments, water used was distilled and purified by the Milli-Q system (Millipore, Mississauga, Canada). ^1^H NMR and ^13^C NMR spectra of final compounds were recorded on a Bruker Ultra-shield 400 MHz Plus spectrometer (Bruker, Rheinstetten, Germany) using TMS as the internal standard (see Supplementary Materials). All chemical shifts are reported in the standard δ notation of parts per million. High-Resolution Mass Spectra were obtained using Waters UPLC Class I/XevoG2Q-Tof (Waters, Milford, MA, USA). 

### 3.1. Synthesis of Compound ***1***, ***2***, ***6***, ***7*** and ***9***

#### 3.1.1. 1-(4-(1,3,2-Dithiarsolan-2-yl) aniline)-2-N-(2,3,4,6-tetra-*O*-acetyl-β-d-glucopyranose-1-yl)-thiourea (**1**)

Compound **9** (200 mg, 0.5136 mmol) and compound **6** (134 mg, 0.5136 mmol) were dissolved in 10 mL anhydrous dichloromethane. It was stirred overnight. The reaction was monitored by TLC (thin-layer chromatography (PE:EA = 2:1, R*_f_* = 0.28)). When the reaction was over, the solvent was removed under reduced pressure. The residue was purified by preparation of thin-layer chromatography with eluant (PE:EA = 1:1). The product was obtained as a white solid (273.6 mg, 81.9%). The product was characterized by IR, ^1^H NMR, ^13^C NMR and HRMS. IR (KBr): 3434 (NH), 2922 (CH_3_ or CH_2_), 2853 (CH_3_ or CH_2_), 1748 (C=O),1626, 1530, 1379, 1227 (C-S), 1034 (C-O-C), 825 (Ph-H) cm^−1^; ^1^H NMR (400 MHz, chloroform-*d*) δ: 7.92 (s, 1H, NH-Ph), 7.66 (d, *J* = 8.0 Hz, 2H, Ph-H), 7.10 (d, *J* = 8.0 Hz, 2H, Ph-H), 6.56 (d, *J*_1,2_ = 8.0 Hz, 1H, anomer-H), 5.74–5.70 (t, *J* = 8.0 Hz, 1H, CH in glucosamine), 5.31–5.26 (t, *J* = 12 Hz, 8 Hz,1H, CH in glucosamine), 4.99–4.94 (t, *J* = 12 Hz, 8 Hz, 1H, CH in glucosamine), 4.86–4.81 (t, *J* = 12 Hz, 8 Hz, 1H, CH in glucosamine), 4.29–4.24 (dd, *J* = 12 Hz, 8.0 Hz, 1H, O-CH_2_), 4.03 (d, *J* = 12 Hz, 1H, O-CH_2_), 3.81–3.77 (m, 1H, NH-anomer), 3.37–3.29 (m, 2H, CH_2_-S), 3.15–3.07 (m, 2H, CH_2_-S), and 2.01–1.94 (m, 12H, CH_3_C=O). ^13^C NMR (101 MHz, chloroform-*d*) δ: 181.1(C=S), 170.0(C=O), 169.6(C=O), 168.8 (C=O), 168.6 (C=O), 142.8 (NH-Ph), 134.9 (AS-Ph), 131.5 (CH in Ph), 123.8 (CH in Ph), 82.3 (anomeric CH), 72.7 (CH in glucoamine), 71.6 (CH in glucoamine), 69.5 (CH in glucoamine), 67.2 (CH in glucoamine), 60.6 (CH_2_-OAc), 41.0 (CH_2_-As), 19.8 (CH_3_ in acetyl), 19.7 (CH_3_ in acetyl), and 19.6 (CH_3_ in acetyl); HRMS: [M + H]^+^: C_23_H_30_AsN_2_O_9_S_3_, calculated: 649.0324, and found: 649.0306.

#### 3.1.2. 1-(4-(1,3,2-Dithiarsinan-2-yl) aniline)-2-N-(2,3,4,6-tetra-*O*-acetyl-β-d-glucopyranose-1-yl)-thiourea (**2**)

Compound **9** (200 mg, 0.5136 mmol) and compound **7** (140.4 mg, 0.5136 mmol) were dissolved in 10 mL anhydrous dichloromethane. This was stirred overnight. The reaction was monitored by TLC (thin-layer chromatography (PE:EA = 2:1, R*_f_* = 0.48)). When the reaction was over, the solvent was removed under reduced pressure. The residue was purified by preparation of thin-layer chromatography with eluant (PE:EA = 1:1). The product was obtained as a white solid (243 mg, 71.5%). The product was characterized by IR, ^1^H NMR, ^13^C NMR and HRMS. IR (KBr): 3493 (NH), 2924 (CH_3_ or CH_2_), 1746 (C=O), 1631, 1539, 1378, 1214 (C-S), 1043 (C-O-C), 801 (Ph-H) cm^−1^; ^1^H NMR (400 MHz, chloroform-*d*) δ: 7.99 (s, 1H, NH-Ph), 7.93 (d, *J* = 8.0 Hz, 2H, Ph-H), 7.24 (d, *J* = 8.0 Hz, 2H, Ph-H), 6.69 (d, *J*_1,2_ = 8.0 Hz, 1H, anomer-H), 5.75–5.70 (t, *J* = 12 Hz, 8.0 Hz, 1H, CH in glucoamine), 5.33–5.28 (t, *J* = 12 Hz, 8.0 Hz, 1H, CH in glucoamine), 5.01–4.96 (t, *J* = 12 Hz, 8.0 Hz, 1H, CH in glucoamine), 4.89–4.84 (t, *J* = 12 Hz, 8.0 Hz, 1H, CH in glucoamine), 4.32–4.28 (dd, *J* = 12 Hz, 4.0 Hz, 1H, O-CH_2_), 4.08–4.02 (m, 1H, O-CH_2_), 3.83–3.79 (m, 1H, NH-anomer), 2.84–2.75 (m, 2H, CH_2_-S), 2.67–2.61 (m, 2H, CH_2_-S), 2.17–2.07 (m, 1H, H-methylene), 2.01–1.94 (m, 12H, CH_3_C=O), 1.91–1.87 (m, 1H, H-methylene). ^13^C NMR (101 MHz, chloroform-*d*) δ: 181.1 (C=S), 170.0 (C=O), 169.6 (C=O), 168.8 (C=O), 168.6 (C=O), 137.8 (NH-Ph), 134.9 (AS-Ph), 133.3 (CH in Ph), 124.6 (CH in Ph), 82.3 (anomeric CH), 72.7 (CH in glucoamine), 71.6 (CH in glucoamine), 69.5 (CH in glucoamine), 67.2 (CH in glucoamine), 60.6 (CH_2_-OAc), 27.1 (CH_2_-As), 24.8 (CH_2_-As), 24.7 (CH_2_), 19.8 (CH_3_ in acetyl), 19.7 (CH_3_ in acetyl), 19.6 (CH_3_ in acetyl); HRMS: [M + H]^+^: C_24_H_32_AsN_2_O_9_S_3_, calculated: 663.0480, and found: 663.0461.

#### 3.1.3. 4-(1,3,2-Dithiarsolan-2-yl) aniline (**6**) [24,26]

Compound **6** was synthesized with a yield of 87% as described in previous literature. It was characterized by IR, ^1^H NMR, ^13^C NMR and HRMS. IR (KBr): 3434 (NH), 3410 (NH), 2959 (CH_3_ or CH_2_), 2926 (CH_3_ or CH_2_), 2855 (CH_3_ or CH_2_), 1618 (Ph), 1579 (Ph), 1408 (Ph), 1276 (C-S), 1060 (C-O-C), 812 (C-H in Ph) cm^−1^; ^1^H NMR (400 MHz, CDCl_3_) δ: 7.45–7.43 (m, 2H, aromatic-H near As), 6.69–6.67 (m, 2H, aromatic-H near NH_2_), 3.80 (br, 2H, NH_2_), 3.40–3.33 (m, 2H, CH_2_), 3.27–3.21 (m, 2H, CH_2_). ^13^C NMR (101 MHz, CDCl_3_) δ: 147.6 (NH_2_-C-Ph), 132.1 (CH of Ph near As), 131.3 (As-C-Ph), 114.9 (CH of Ph near NH_2_), 41.6 (CH_2_), 30.91 (CH_2_). 

#### 3.1.4. 4-(1,3,2-Dithiarsinan-2-yl) aniline **7** [18]

Compound **4** (1 g, 5.4654 mmol) and propane-1,3-dithiol (615 µL mg, 6.1376 mmol) were dissolved in ethanol (15 mL). The solution was refluxed under N_2_ for 5 h. It was monitored by TLC (PE: EA = 4:1, R*_f_* = 0.45). The reaction was cooled to room temperature. The solution was evaporated under reduced pressure to concentrated residue. Then, it was transferred to a refrigerator overnight. It was filtered. The filtered cake was washed with 5 mL cold ethanol twice. Compound **7** was obtained as an off-white solid (1.1761 g, 78.8%). It was characterized by IR, ^1^H NMR, ^13^C NMR and HRMS. IR (KBr): 3435 (NH), 2924 (CH_2_), 2878 (CH_2_), 2853 (CH_2_), 1620 (C=C in Ph), 1588 (C=C in Ph), 1403 (C=C in Ph), 1295 (C-S), 1266 (C-S), 1076 (C-O-C), 812 (C-H in Ph) cm^−1^; ^1^H NMR (400 MHz, CDCl_3_) δ: 7.66–7.64 (d, 2H, *J* = 8.0 Hz, CH at Ph near As), 6.79–6.77 (d, 2H, *J* = 8.0 Hz, CH at Ph near NH_2_), 3.85 (br, 2H, NH_2_), 2.93–2.87 (m, 2H, As-CH_2_), 2.74–2.68 (m, 2H, As-CH_2_), 2.19–2.10 (m, 1H, H of CH_2_), 1.97–1.90 (m, 1H, H of CH_2_). ^13^C NMR (101 MHz, CDCl_3_) δ: 146.5 (NH_2_-C-Ph), 132.8 (CH of As-Ph), 124.7 (NH_2_-CH-Ph), 114.6 (As-C-Ph), 27.7 (As-CH_2_), 25.4 (As-CH_2_).

#### 3.1.5. 2,3,4,6-Tetra-*O*-acetyl-β-d-glucopyranosyl isothiocyanate (**9**) [30]

Potassium thiocyanate (473 mg, 4.863 mmol), a 4Å molecular sieve (3.48 g) and tetrabutylammonium bromide (784 mg, 2.432 mmol) were added to 30 mL anhydrous acetonitrile. It was stirred under N_2_ at room temperature for 3 h. 2,3,4,6-tetra-*O*-acetyl-α-d-glucopyranosyl bromide **8** (1000 mg, 2.432 mmol) was added to the solution. The reaction was heated to reflux under N_2_ for 4 h. It was monitored by thin-layer chromatography with eluant (PE:EA = 3:1). When the reaction was over, the solution was cooled to the room temperature. The solvent was removed under reduced pressure. The residue was purified by silica gel chromatography with eluant (PE:EA = 3:1). The product was obtained as a off-white solid with a yield of 76.3% (690.8 mg). It was characterized by IR, ^1^H NMR, ^13^C NMR and HRMS. IR (KBr): 3434, 2985 (CH_3_), 2940 (CH_3_), 2883 (CH_3_), 2113 (SCN), 1746 (C=O), 1626, 1383 (C-H in CH_3_), 1237 (C-S), 1219 (C-S), 1061 (C-O-C), 1030 (C-O-C), 911 cm^−1^; ^1^H NMR (400 MHz, CDCl_3_) δ: 5.24–5.19 (t, *J* = 12.0 Hz, 8.0 Hz, 1H, CH in glucoamine), 5.14–5.12 (d, *J*_1,2_ = 8.0 Hz, 1H, anomer-H), 5.11–5.09 (t, *J* = 4 Hz, 1H, CH in glucoamine), 5.03 (d, *J*_1,2_ = 8 Hz, 1H, CH in glucoamine), 4.27–4.22 (m, 2H, CH_2_-O), 3.77–3.73 (m, 1H, CH in glucoamine), 2.12–2.11 (m, 6H, CH_3_ in acetyl), 2.05–2.03 (m, 6H, CH_3_ in acetyl).

### 3.2. Cell Lines and Cell Culture *[25]*

HeLa and HepG2 cells were obtained from Stem Cell Bank, Chinese Academy of Sciences. The human colon cancer HCT-116 cells and human embryonic kidney cells 293T were purchased from the American Type Culture Collection (ATCC). Cells were cultured in high-glucose DMEM (Gibco, Carlsbad, CA, USA) supplemented with 10% (*v*/*v*) FBS (Gibco, Grand Island, NE, USA) and 1% (*v*/*v*) penicillin–streptomycin (PS) (Gibco, Grand Island, NE, USA), and maintained at 37 °C under 5% CO_2_ humidified atmosphere. Only cells in logarithmic phase were used in all following experiments.

### 3.3. MTT Assay *[25]*

The cytotoxicity of compounds **1**, **2**, **6** and **7** in Hela, HepG2, HCT 116 and 293T cells was evaluated by MTT assay. Cells were seeded onto a 96-well plate (Corning, Corning, NY, USA) with a density of 3000 cells/well for 24 h in high-glucose DMEM (Gibco, Carlsbad, CA, USA) supplemented with 10% (*v*/*v*) FBS (Gibco, Grand Island, NE, USA) and 1% (*v*/*v*) penicillin–streptomycin (PS) (Gibco, Grand Island, NE, USA), and then treated with tested compounds at various concentrations for 72 h. MTT solution was added to each well for 4 h, and the absorbance was measured at 495 nm by using Varioskan Flash Multimode Reader (Thermo, Waltham, MA, USA). The values of IC_50_ were calculated by GraphPad Prism Software 9.0.

## 4. Conclusions

In this investigation, to avoid the form mixture of α/β anomers, the strategy of 2-acetyl’s neighboring group participation was used to lock the β anomeric configuration of 2,3,4,6-tetra-*O*-acetyl-β-d-glucopyranosyl isothiocyanate **9** based on 2,3,4,6-tetra-*O*-acetyl-α-d-glucopyranosyl bromide through a five-membered glycosyl oxocarbenium ion transition state. In the following steps, compounds **6** and **7** reacted with **9** involving a nucleophilic attack to form our target compounds **1** and **2** at room temperature, while their anomeric configurations were still β. The results of ^1^H NMR identified these cases. 1-(4-(1,3,2-dithiarsinan-2-yl)aniline)-2-*N*-(2,3,4,6-tetra-*O*-acetyl-β-d-glucopyranos-1-yl)-thiourea **2** can increase the selectivity of human colon cancer cells HCT-116 (0.82 ± 0.06 μM vs. 1.82 ± 0.07μM) to human embryonic kidney 293T cells (1.38 ± 0.01 μM vs. 1.22 ± 0.06 μM) from 0.67 to 1.68, while the cytotoxicity towards normal cells (293T) was still maintained, which was less than what we expected. These results suggested a new method for the discovery and development of arsenic-containing compounds as novel chemotherapeutics for the treatment of solid tumors. 

## Figures and Tables

**Table 1:** The cytotoxicity of compounds **1** and **2** on 293T, HCT116, Hela and HepG2 cells.

IC_50_ (μM)				
	1	2	6	7
293T	3.14 ± 0.06	1.38 ± 0.01	4.06 ± 0.07	1.22 ± 0.06
Hela	14.4 ± 0.23	11.05 ± 0.17	6.49 ± 0.40	4.52 ± 0.68
HepG2	7.62 ± 0.10	2.81 ± 0.03	6.11 ± 0.14	3.10 ± 0.37
HCT-116	1.77 ± 0.11	0.82 ± 0.06	1.86 ± 034 ^a^	1.82 ± 0.07
Selectivity index ^b^	1.77	1.68	2.18	0.67

^a^ [18]. ^b^ Selectivity index: the IC_50_ against HCT116 over the IC_50_ against 293T.

## Data Availability

The data presented in this study are available upon request from the corresponding author.

## Supplementary Materials

The following supporting information can be downloaded at: https://www.mdpi.com/article/10.3390/molecules29122850/s1, Figure S1: ^1^H NMR of Compound **8;** Figure S2: ^1^H NMR of Compound **9;** Figure S3: ^1^H NMR of Compound **6**; Figure S4: ^13^C NMR of Compound **6**; Figure S5: ^1^H NMR of Compound **7**; Figure S6: ^13^C NMR of Compound **7**; Figure S7: ^1^H NMR of Compound **1**; Figure S8: ^13^C NMR of Compound **1**; Figure S9: HRMS of Compound **1**; Figure S10: ^1^H NMR of Compound **2**; Figure S11: ^13^C NMR of Compound **2**; Figure S12: HRMS of Compound **2**; Figure S13: IR of Compound **1**; Figure S14: IR of Compound **2**; Figure S15: IR of Compound **6**; Figure S16: IR of Compound **7**; Figure S17: IR of Compound **9**; Figure S18: IC_50_ of Compound **1**, **2** and **7** for HCT-116; Figure S19: IC_50_ of Compound **1**, **2**, **6** and **7** for 293T; Figure S20: IC_50_ of Compound **6** for Hela and HepG2; Figure S21: IC_50_ of Compound **7** for Hela and HepG2; Figure S22: IC_50_ of Compound **1** and **2** for Hela; Figure S23: IC_50_ of Compound **1** and **2** for HepG2.
