# Supplementary material for: Design and Synthesis of Thiourea-Conjugating Organic Arsenic D-Glucose with Anticancer Activities
Source: Molecules. 2024 Jun 14;29(12):2850. doi: 10.3390/molecules29122850 (PMC11206549; doi:10.3390/molecules29122850)
Supplement: Supplementary file 1 [file molecules-29-02850-s001.zip › molecules-3021665-supplementary.pdf]

# Design and synthesis of thiourea-conjugating organic arsenic D-glucose with anticancer activities

Boqiao Fu [1,*], Wenxuan Liu[1], Yufeng Wang[1], Guorui Li[2], Yingsha Wang[3], Xinyuan Huang[4], Hongan Shi[4] and Caiqin Qin[1]

[1]College of Chemistry and Materials Science, Hubei Engineering University, 432000, Hubei, P.R. China

[2]Hunan Provincial Key Laboratory of the Research and Development of Novel Pharmaceutical Preparations, the "Double-First Class" Application Characteristic Discipline of Hunan Province (Pharmaceutical Science), Changsha Medical University, Changsha, 410219, P. R. China

[3] State Key Laboratory for Chemo/Bio-Sensing and Chemometrics, College of Chemistry and Chemical Engineering, School of Biomedical Sciences, Hunan University, Changsha, 410082, P. R. China

[4] Hubei Key Laboratory of Quality Control of Characteristic Fruits and Vegetables, College of Life and Technology, Hubei Engineering University, Hubei, 432000, P.R. China

*Correspondence: B. F. fuboqiao@126.com, College of Chemistry and Materials Science, Hubei Engineering University, 432000, Hubei, P.R. China. Tel: +86-712-2345464.

## Supporting Information

### Table of contents

Figure S1. $^{1}$H NMR of Compound **8** ......S1

Figure S2. $^{1}$H NMR of Compound **9** ......S1

Figure S3. $^{1}$H NMR of Compound **6** ......S2

Figure S4. $^{13}$C NMR of Compound **6** ......S2

Figure S5. $^{1}$H NMR of Compound **7**......S3

Figure S6. $^{13}$C NMR of Compound **7**......S3

Figure S7. $^{1}$H NMR of Compound **1**......S4

Figure S8. $^{13}$C NMR of Compound **1**......S4

Figure S9. HRMS of Compound **1**......S5

Figure S10. $^{1}$H NMR of Compound **2**......S5

Figure S11. $^{13}$C NMR of Compound **2**......S6

Figure S12. HRMS of Compound **2**......S6

Figure S13. IR of Compound **1**......S7

Figure S14. IR of Compound **2**......S7

Figure S15. IR of Compound **6**......S8

Figure S16. IR of Compound **7**......S8

Figure S17. IR of Compound **9**......S9

Figure S18. $IC_{50}$ of Compound **1**, **2** and **7** for HCT-116......S9

Figure S19. $IC_{50}$ of Compound **1**, **2**, **6** and **7** for 293T......S10

Figure S20. $IC_{50}$ of Compound **6** for Hela and HepG2......S10

Figure S21. $IC_{50}$ of Compound **7** for Hela and HepG2......S11

Figure S22. $IC_{50}$ of Compound **1** and **2** for Hela......S11

Figure S23. $IC_{50}$ of Compound **1** and **2** for HepG2......S12

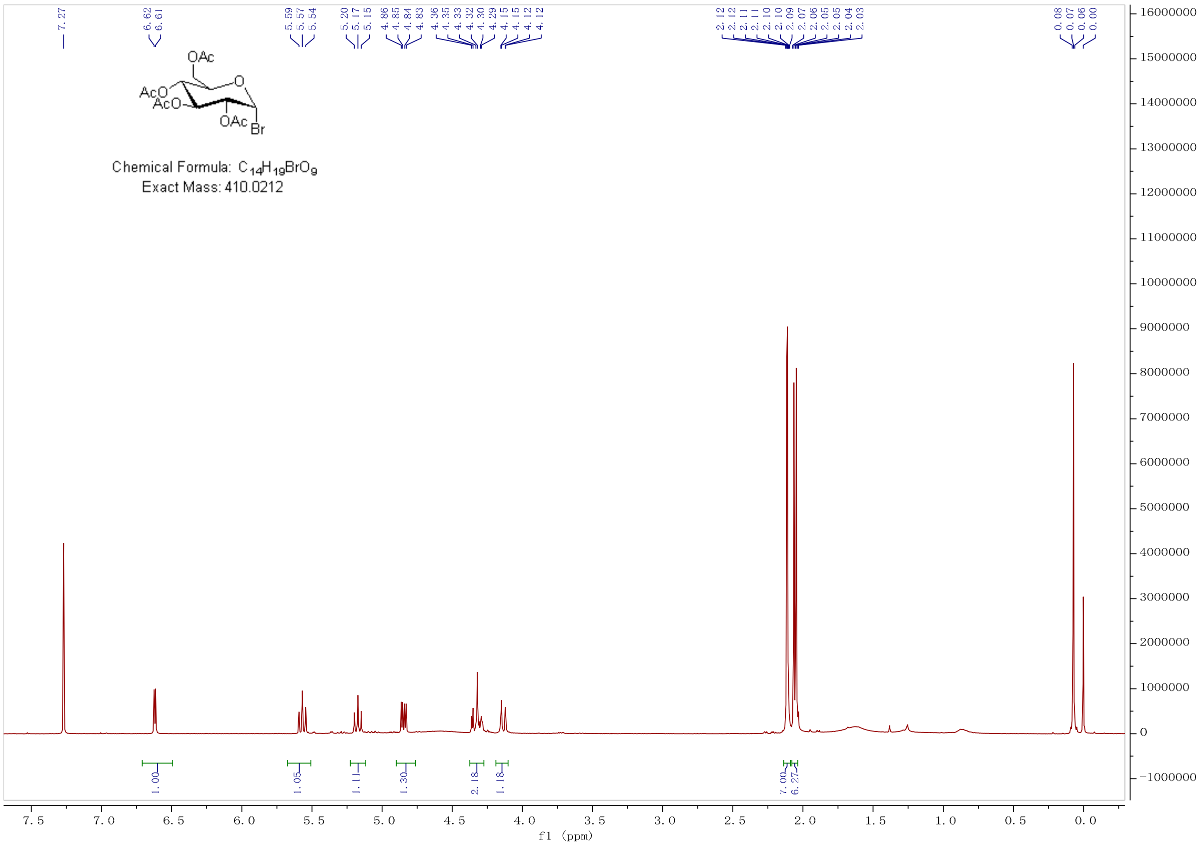


**Figure S1:** $^{1}$H NMR of Compound **8** (400 MHz, $CDCl_3$).

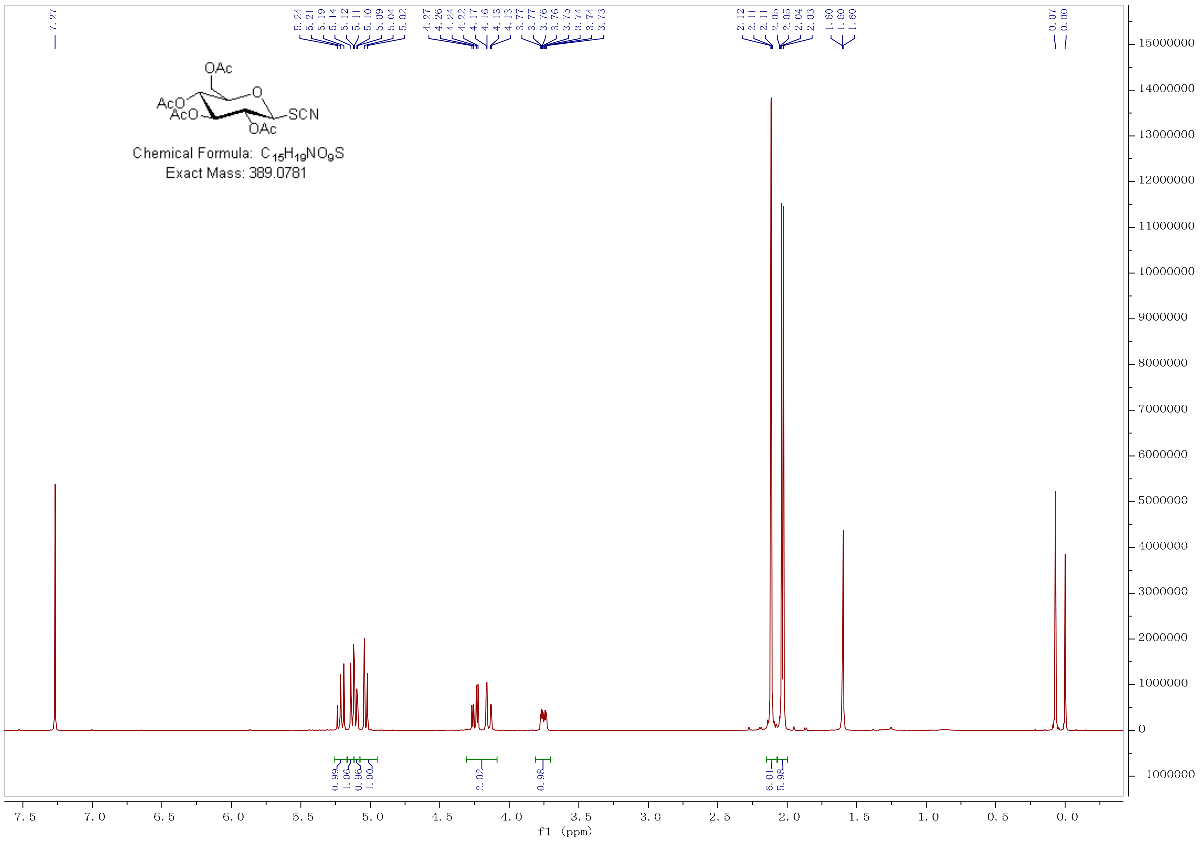


**Figure S2:** $^{1}$H NMR of Compound **9** (400 MHz, $CDCl_3$).

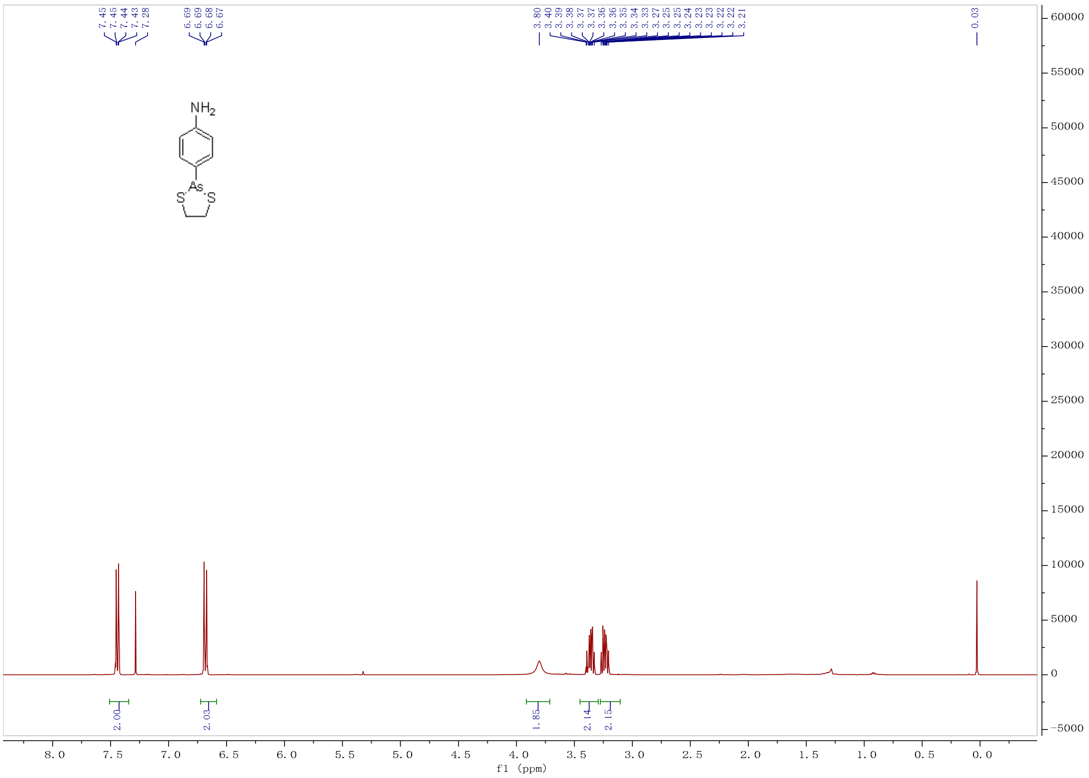


**Figure S3:** ¹H NMR of Compound **6** (400 MHz, CDCl3).

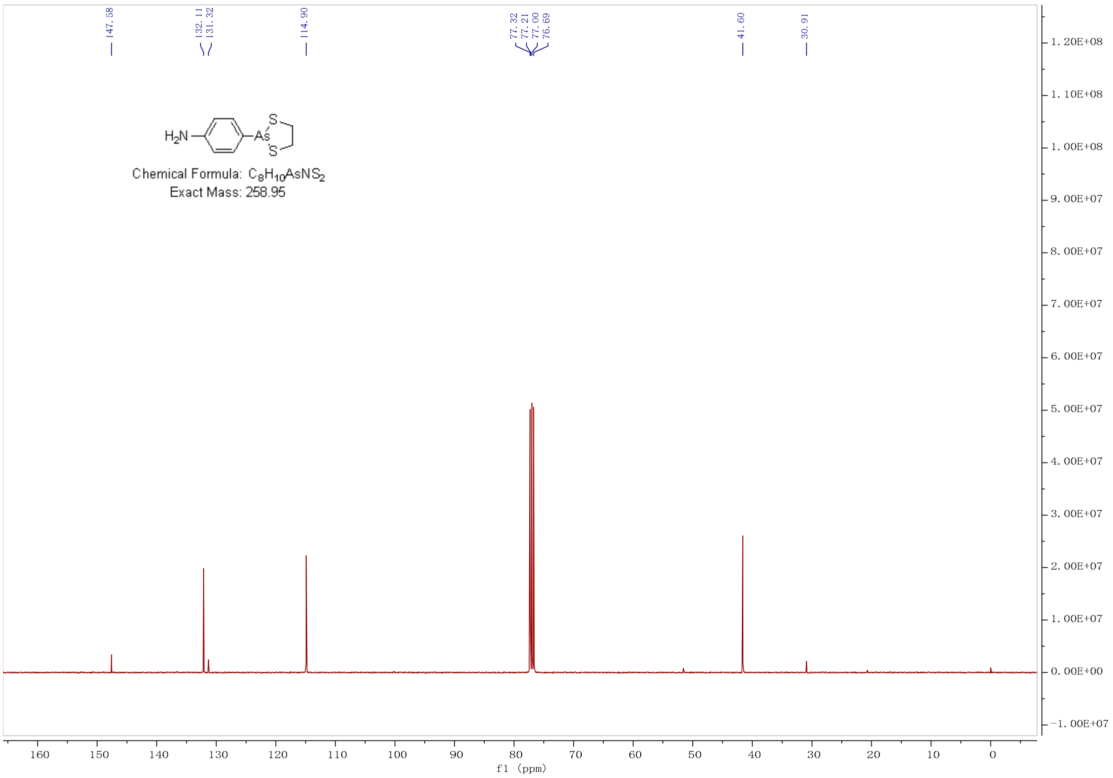


**Figure S4:** ¹³C NMR of Compound **6** (100 MHz, CDCl3).

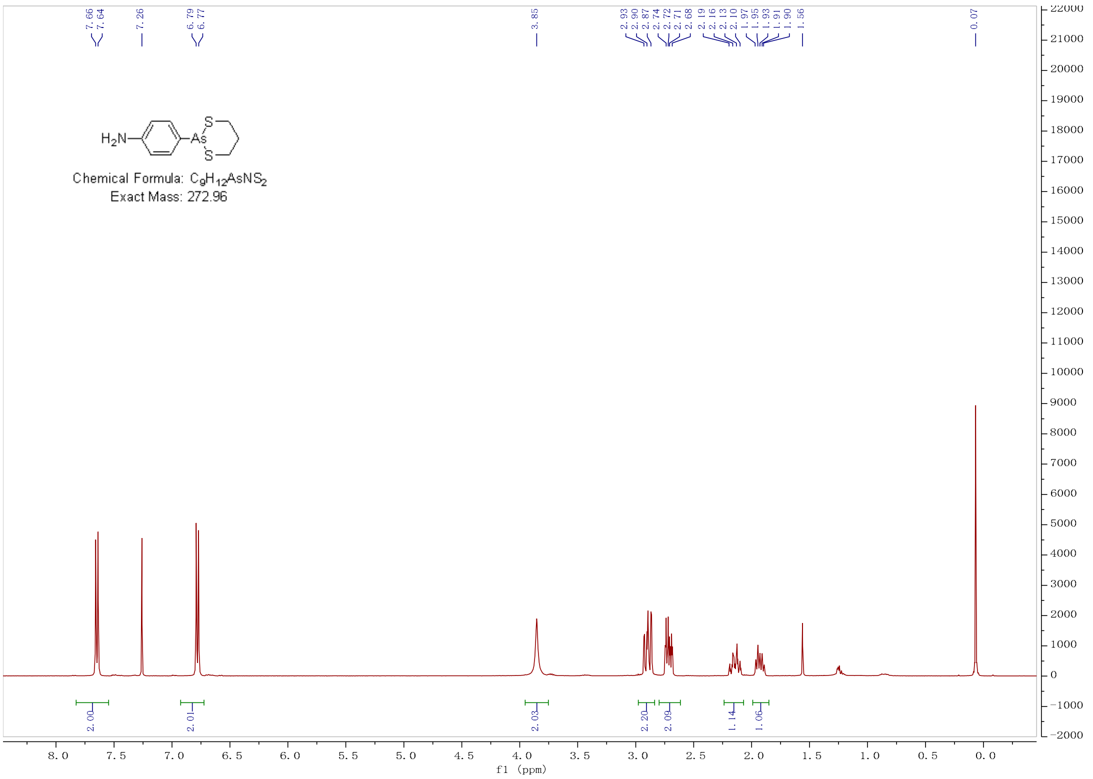


**Figure S5:** $^{1}$H NMR of Compound **7** (400 MHz, $CDCl_3$).

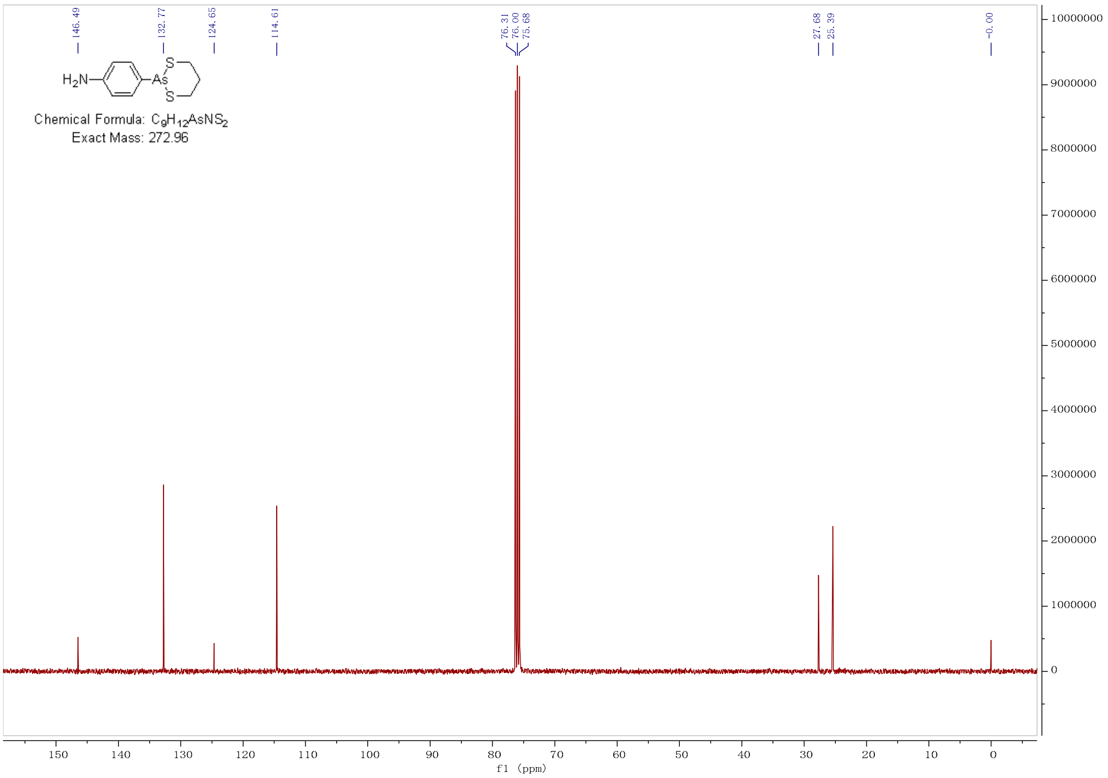


**Figure S6:** $^{13}$C NMR of Compound **7** (100 MHz, $CDCl_3$).

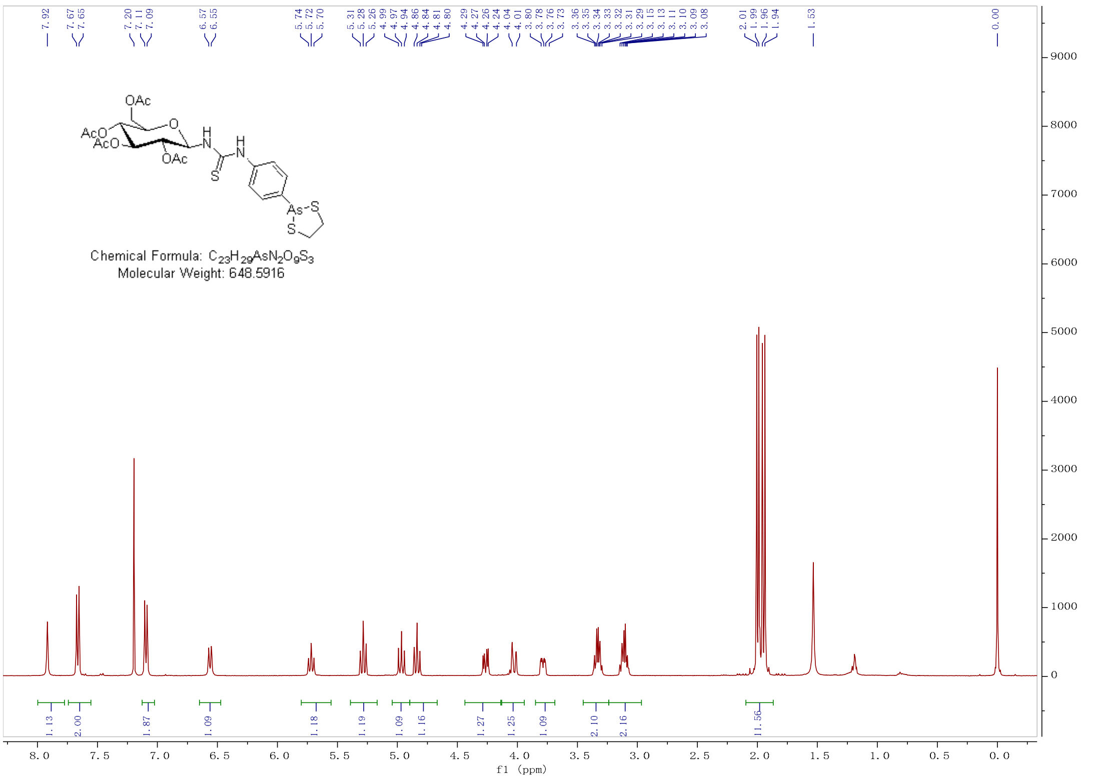

**Figure S7:** $^{1}H$ NMR of Compound **1** (400 MHz, $CDCl_3$).

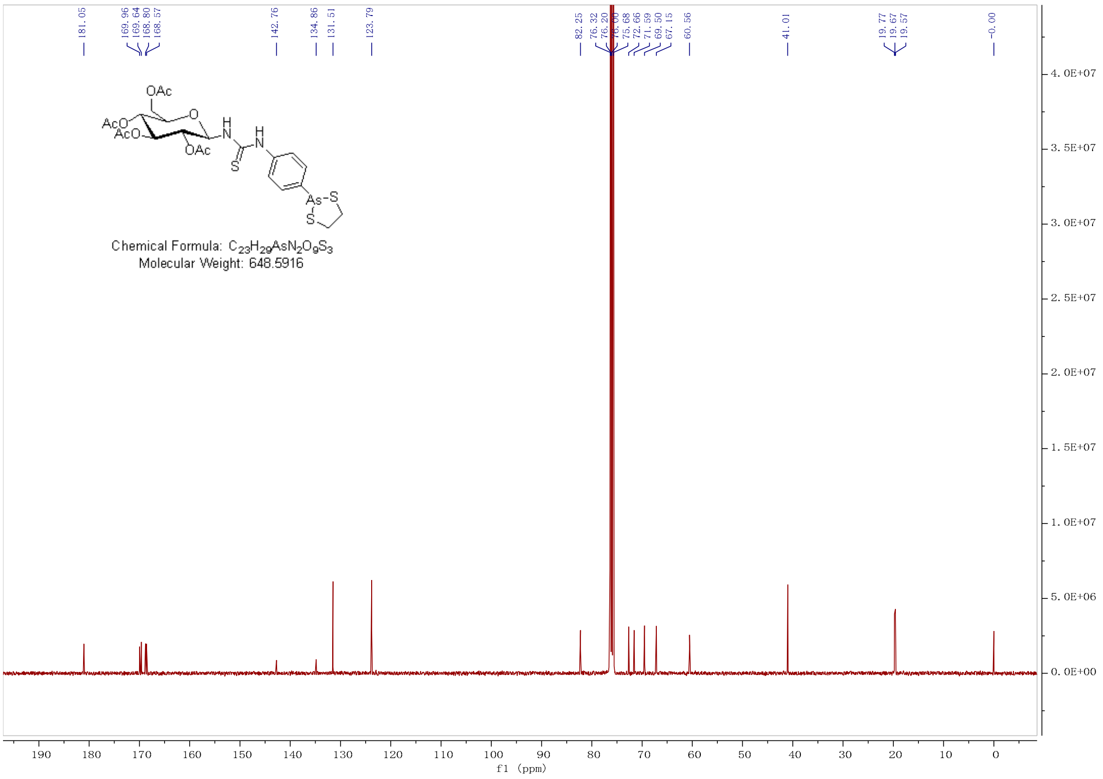

**Figure S8:** $^{13}C$ NMR of Compound **1** (100 MHz, $CDCl_3$).

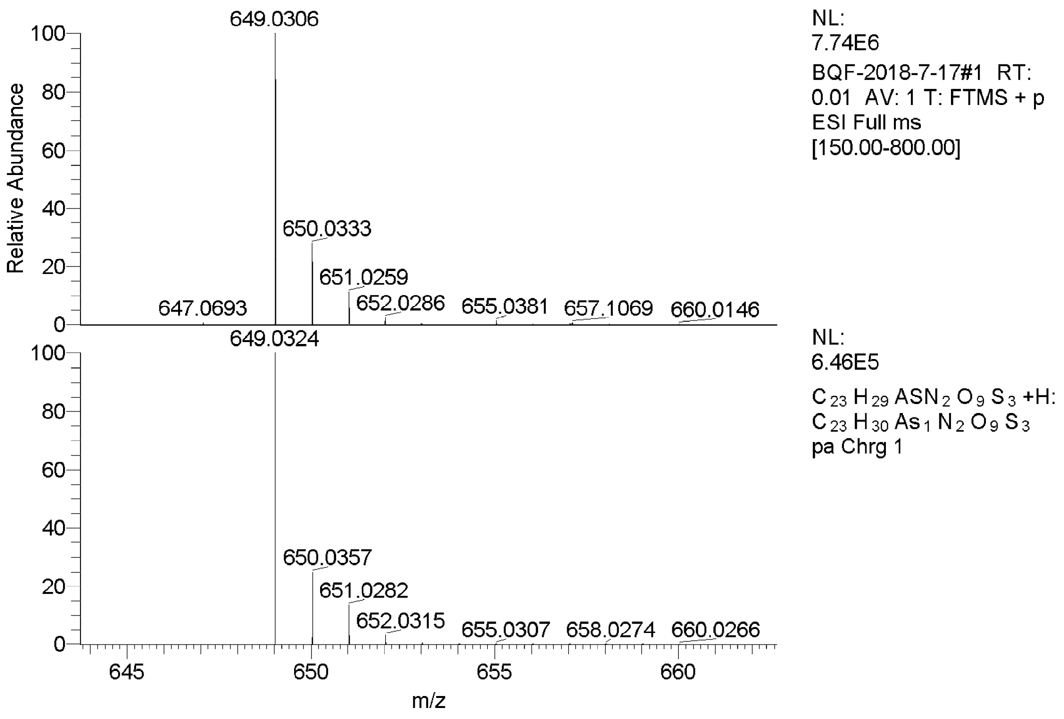


**Figure S9:** HRMS of Compound **1**.

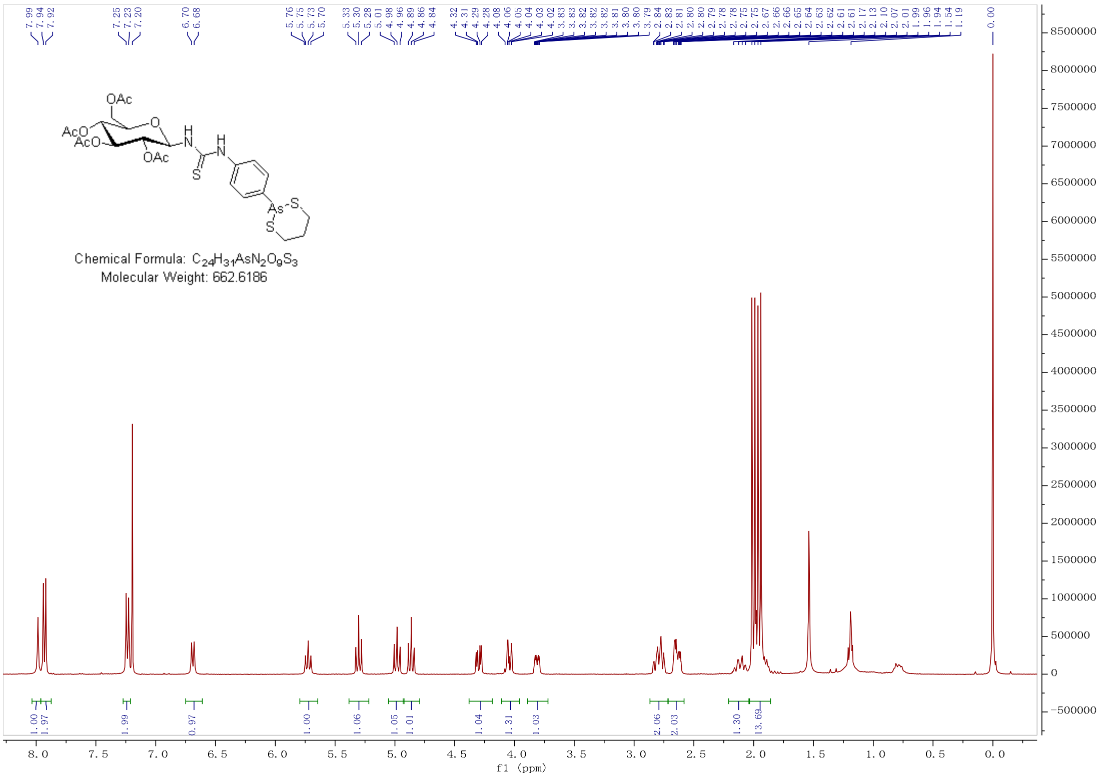


**Figure S10:** $^{1}$H NMR of Compound **2** (400 MHz, $CDCl_3$).

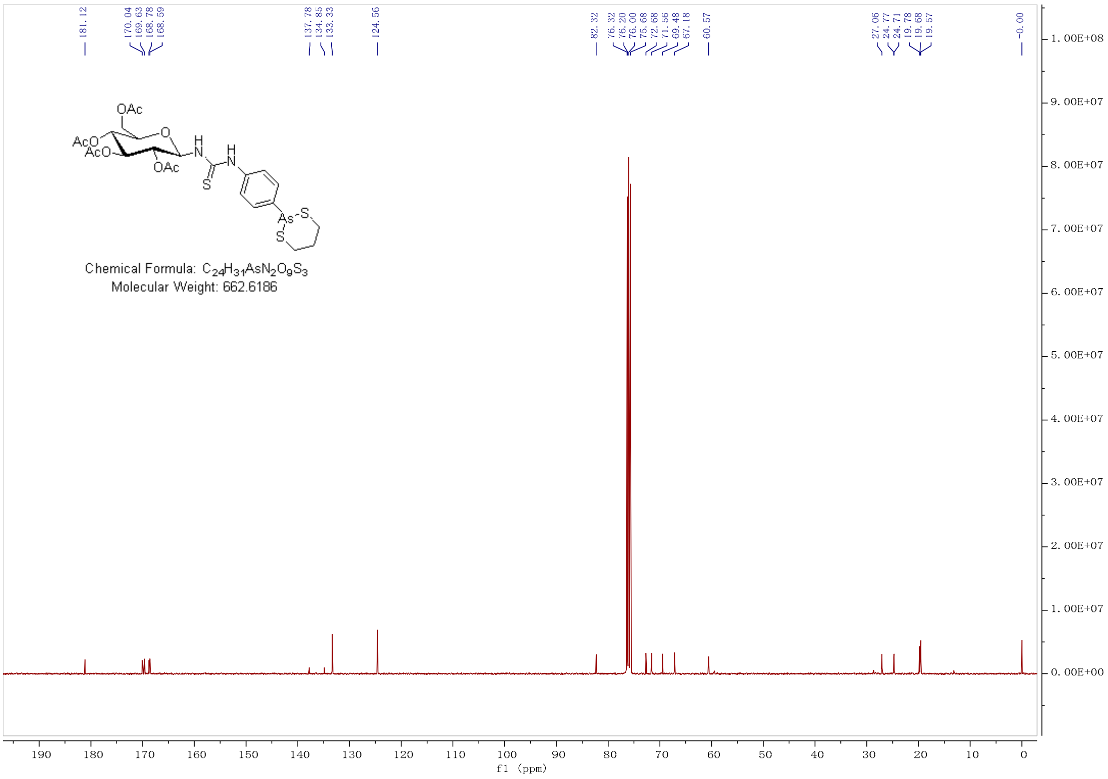


**Figure S11:** $^{13}$C NMR of Compound **2** (100 MHz, $CDCl_3$).

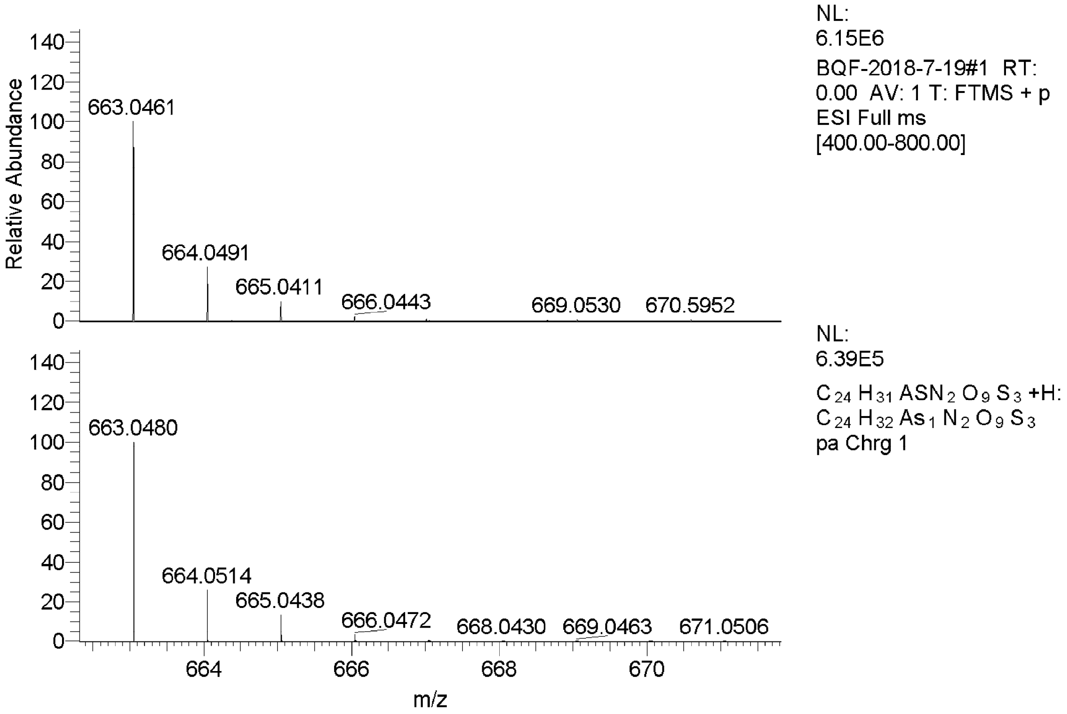


**Figure S12:** HRMS of Compound **2**.

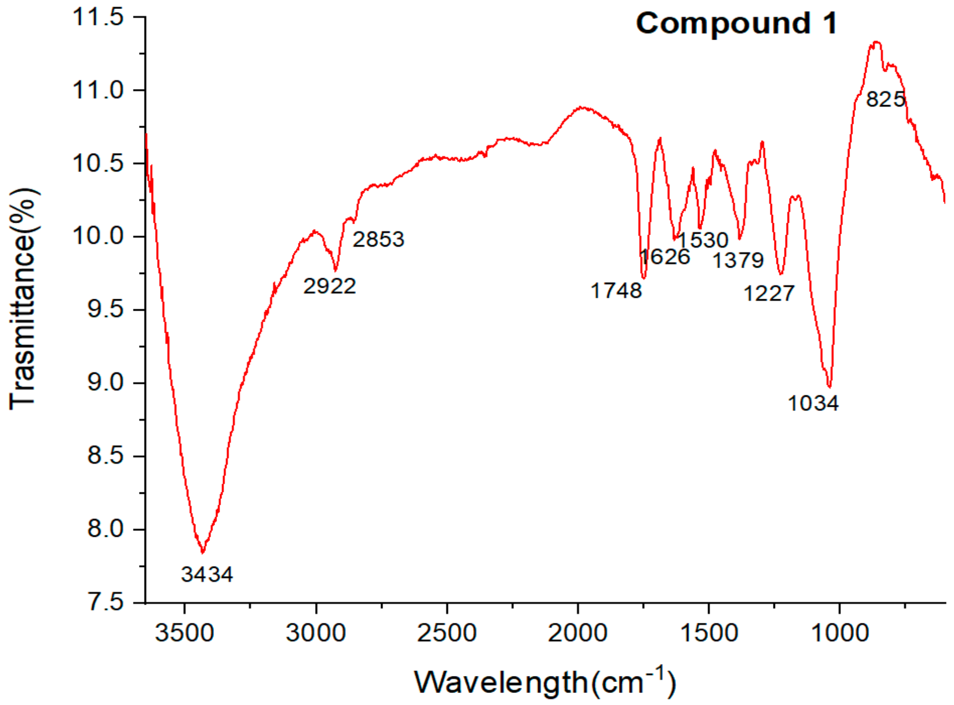


**Figure S13:** IR of Compound **1**(KBr).

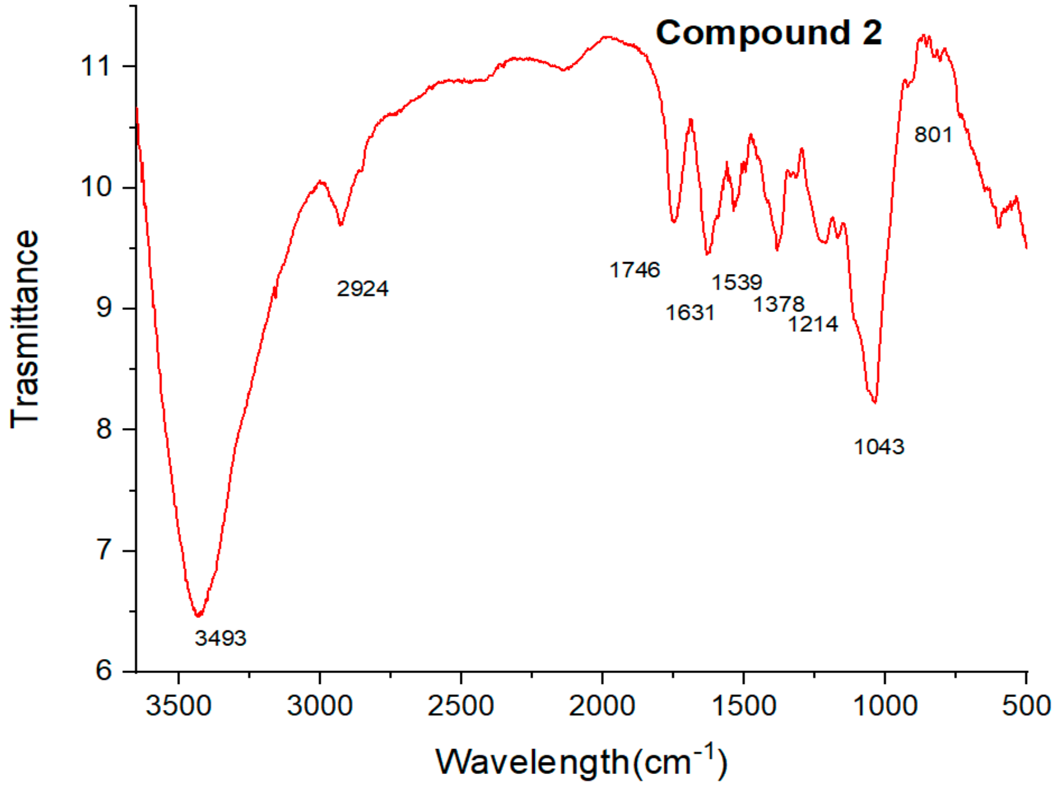


**Figure S14:** IR of Compound **2**(KBr).

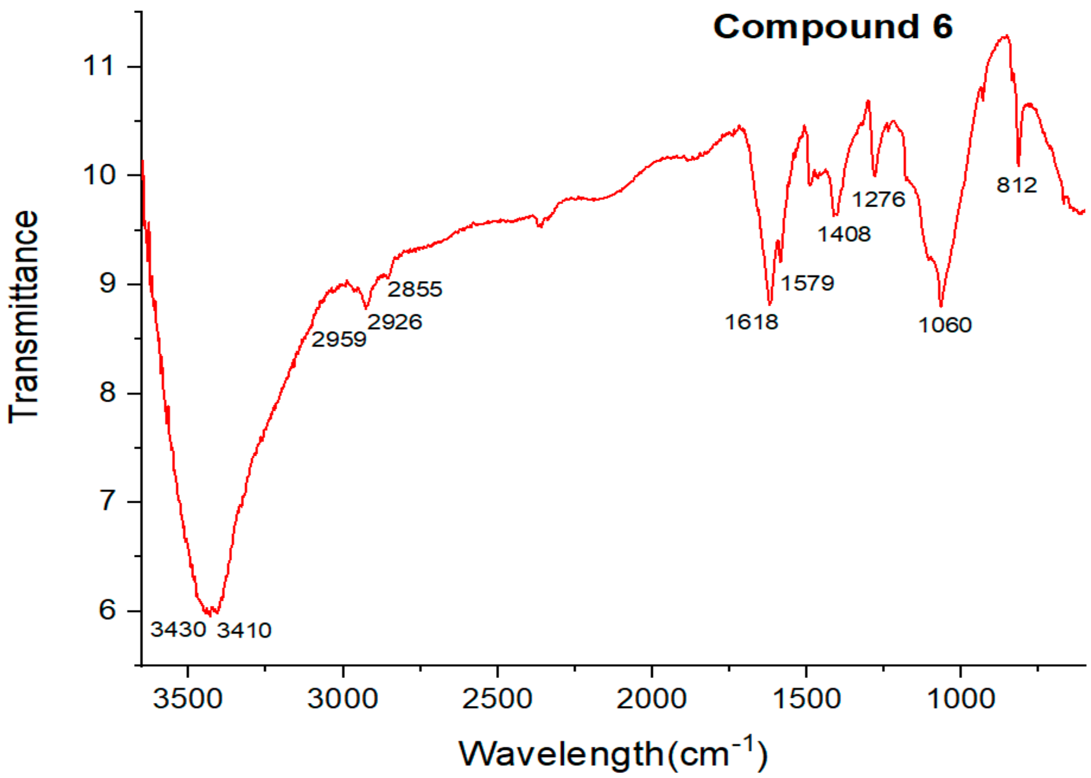


**Figure S15:** IR of Compound **6**(KBr).

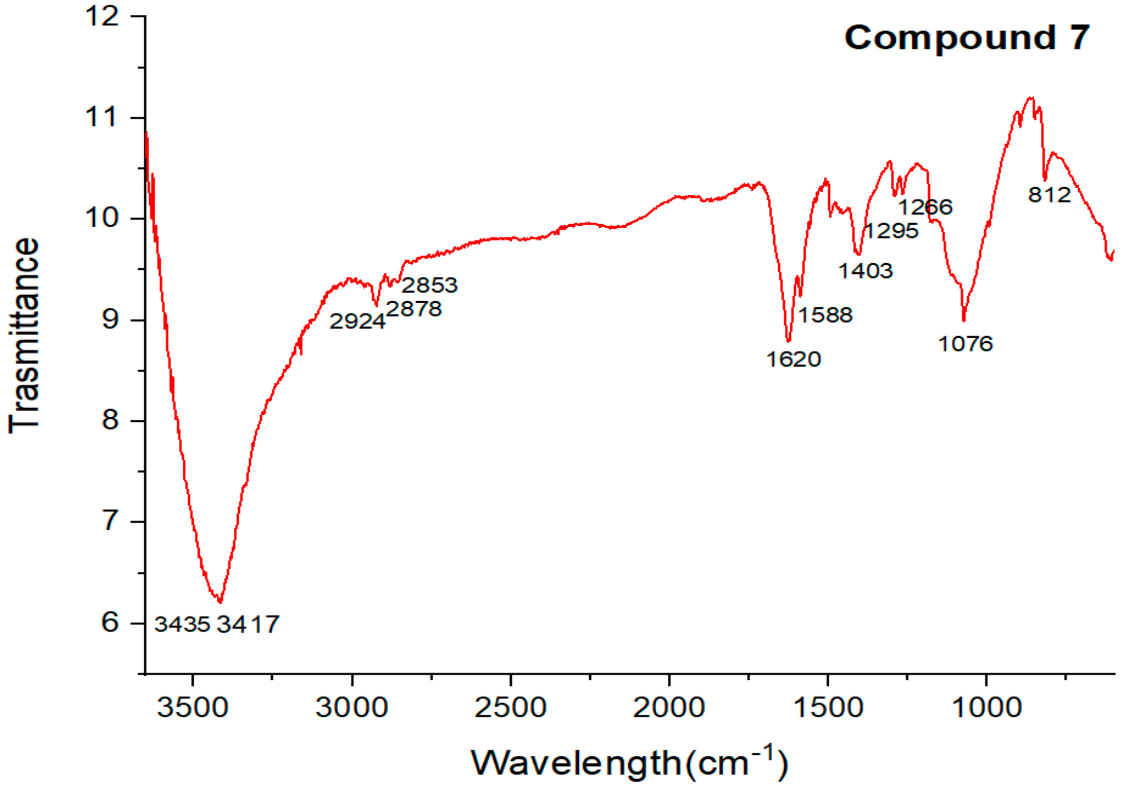


**Figure S16:** IR of Compound **7**(KBr).

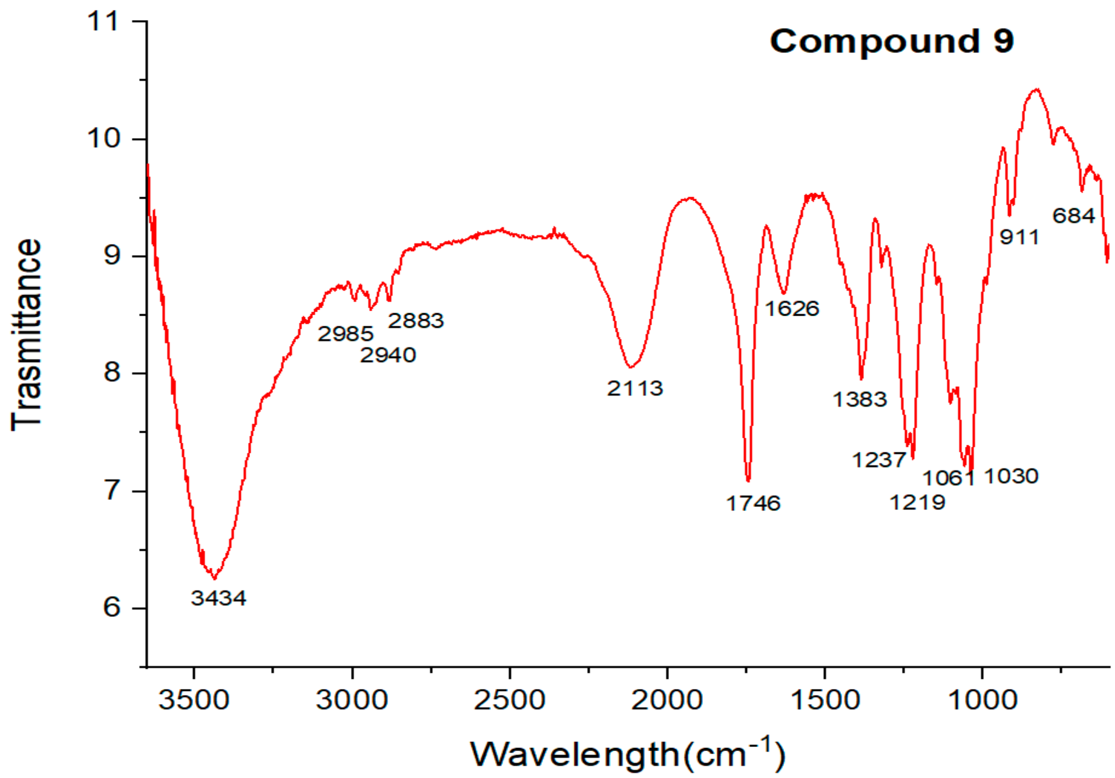


**Figure S17:** IR of Compound **9**(KBr).

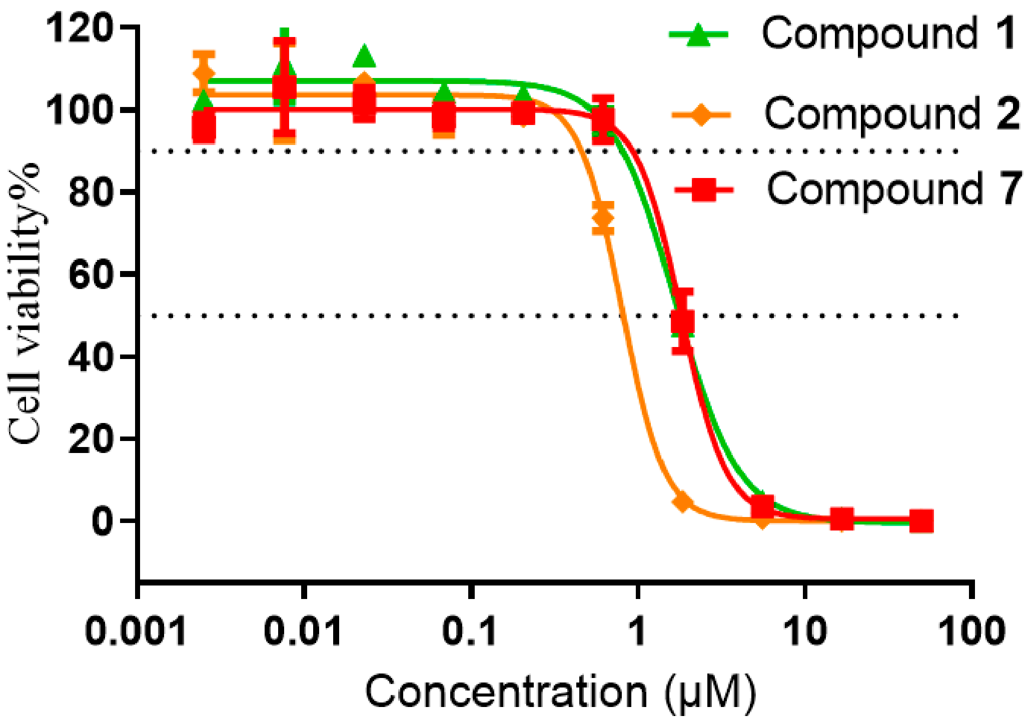


**Figure S18:** $IC_{50}$ of Compound **1**， **2** and **7** for HCT-116.

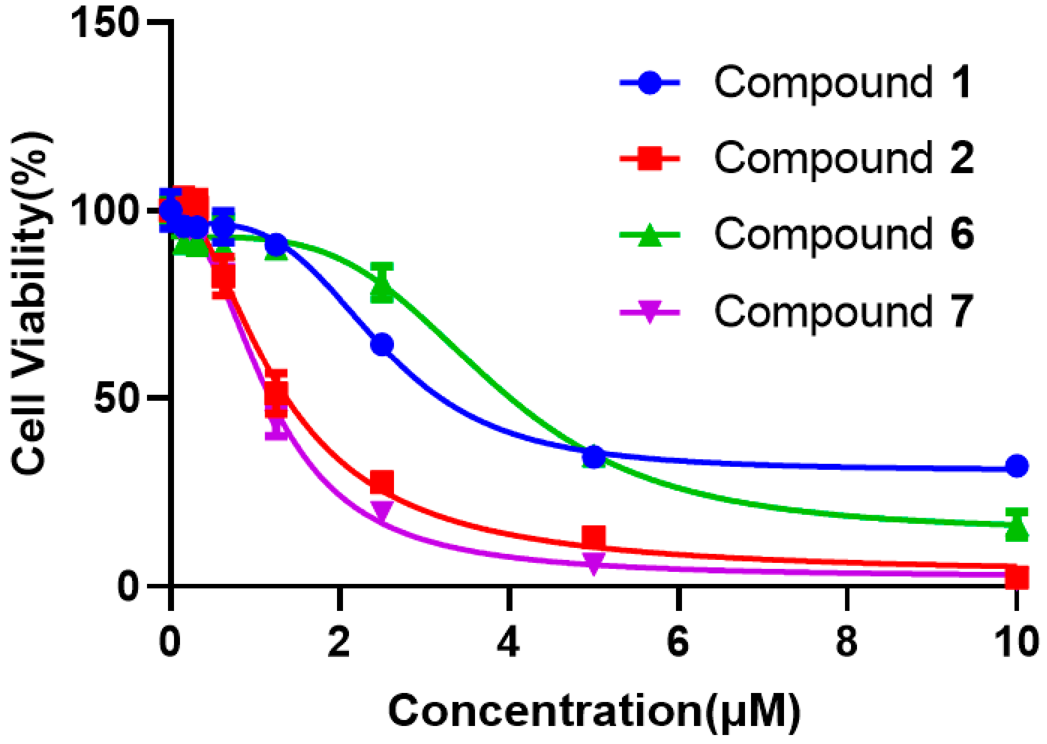


**Figure S19:** $IC_{50}$ of Compound **1**， **2**, **6** and **7** for 293T.

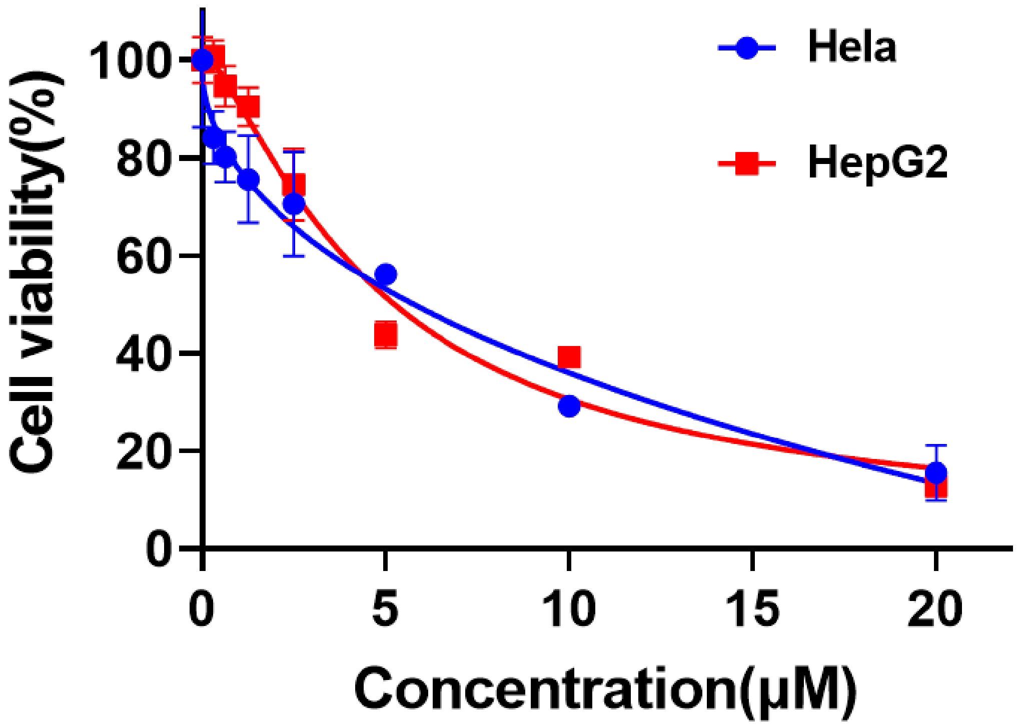


**Figure S20:** $IC_{50}$ of Compound **6** for Hela and HepG2.

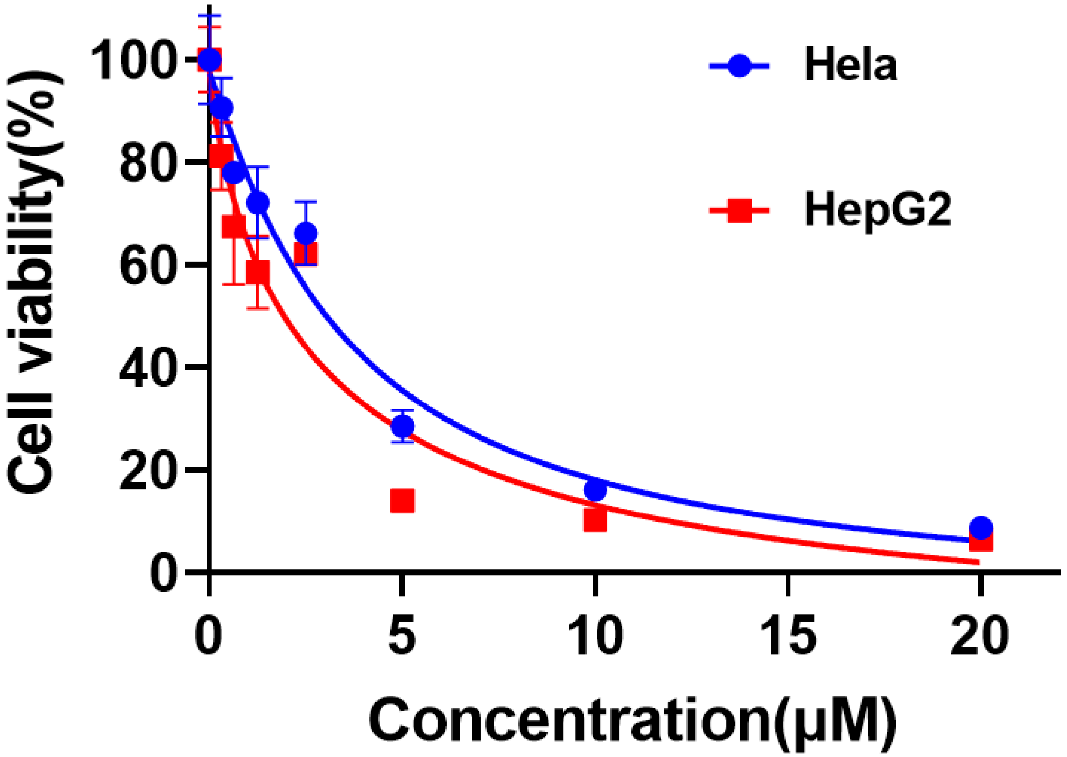


**Figure S21:** $IC_{50}$ of Compound **7** for Hela and HepG2.

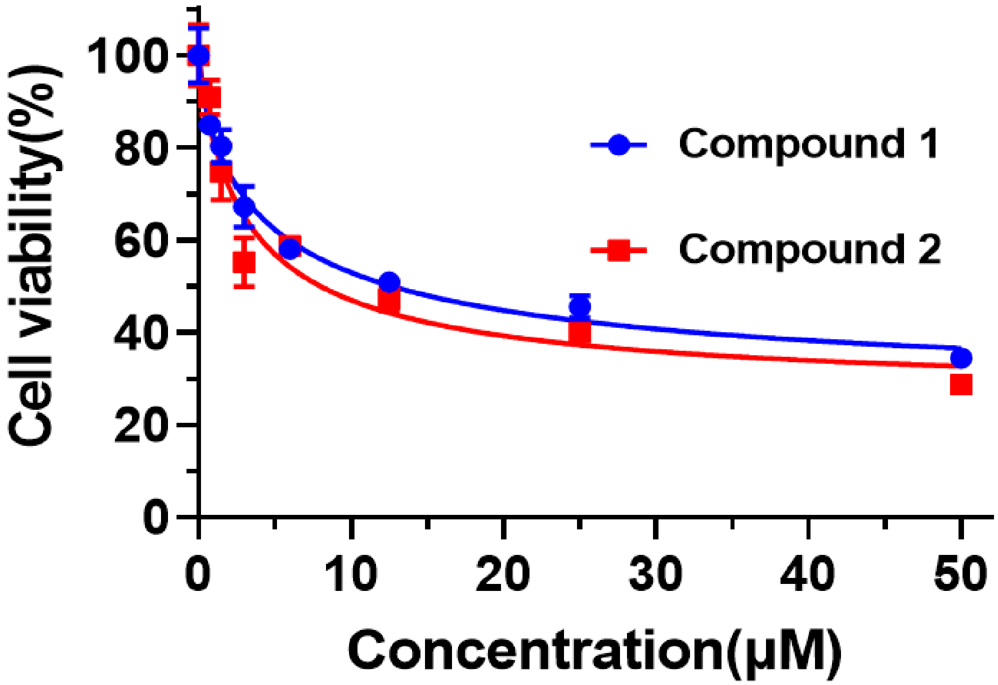


**Figure S22:** $IC_{50}$ of Compound **1**and **2** for Hela.

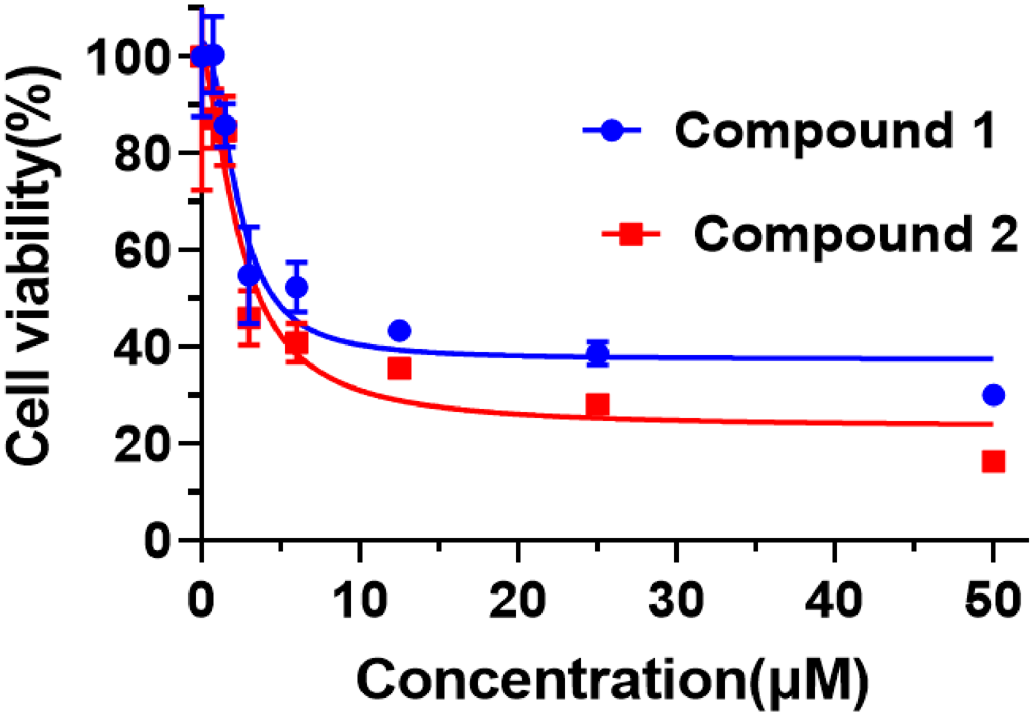


**Figure S23:** $IC_{50}$ of Compound **1**and **2** for HepG2.
